# Common questions and misconceptions of sodium bicarbonate as an ergogenic aid: what does the science really show?

**DOI:** 10.1080/15502783.2026.2724103

**Published:** 2026-09-04

**Authors:** Brandi Antonio, Eli S. Shannon, Darren G. Candow, Lewis A. Gough, Jozo Grgic, William H. Gurton, Lars R. McNaughton, Justin D. Roberts, Bryan Saunders, Antonella V. Schwarz, Jason C. Siegler, S. Andy Sparks, Jeffrey R. Stout, Ecaterina Vasenina, Jose Antonio

**Affiliations:** a Department of Kinesiology and Rehabilitation Sciences, University of Central Florida, Orlando, FL, USA; b Department of Sport and Physical Activity, Edge Hill University, Nutrition, Sport and Exercise Performance Research Group, Ormskirk, UK; c Department of Health and Human Performance, University of Regina, Regina, SK, Canada; d Birmingham City University, Human Performance and Health Laboratory, Centre for Life and Sport Sciences, Birmingham, UK; e Department of Sports Science and Physical Education, The Chinese University of Hong Kong, Hong Kong, China; f Sheffield Hallam University, Food and Nutrition Subject Group, Sheffield Business School, Sheffield, UK; g Consultant in Sport, Nutrition and Exercise Science, Southport, UK; h Anglia Ruskin University, Cambridge Centre for Sport & Exercise Sciences, Faculty of Science & Engineering, Cambridge, UK; i University of São Paulo, Faculty of Medicine, São Paulo, Brazil; j Department of Health Promotion and Clinical Practice, Barry University, Miami Shores, FL, USA; k Arizona State University, College of Health Solutions, Phoenix, AZ, USA; l Maurten AB, Gothenburg, Sweden; m Liverpool John Moores University, Research Institute for Sport and Exercise Sciences, Liverpool, UK; n Department of Health and Human Performance, University of Tampa, Tampa, FL, USA; o Department of Health and Human Performance, Nova Southeastern University, Davie, FL, USA

**Keywords:** Ergogenic aid, extracellular buffer, exercise, performance, supplement

## Abstract

Sodium bicarbonate is an ergogenic extracellular buffering agent that has recently garnered more interest because novel formulations purported to mitigate gastrointestinal symptoms, thereby increasing use with high-profile athletes. The effects of sodium bicarbonate on various aspects of exercise performance have been explored in hundreds of scientific articles. However, questions persist about aspects of its effectiveness and the potential side effects associated with the extracellular buffer. In this review, we provide answers to several questions related to sodium bicarbonate supplementation. These questions covered include: (1) What are sodium bicarbonate's proposed mechanisms for enhancing performance? (2) Does sodium bicarbonate cause gastrointestinal symptoms, and why? (3) What are the different ways in which sodium bicarbonate can be ingested, and how does this affect blood bicarbonate changes and exercise performance? (4) How does sodium bicarbonate affect performance in short-duration exercise (≤10 min)? (5) How does sodium bicarbonate affect performance in long-duration exercise, and should it be used before and during such events? (6) Is there a potential rationale for sodium bicarbonate use at altitude? (7) Is it worth ingesting sodium bicarbonate for exercise in the heat? (8) When is the best time to take sodium bicarbonate before exercise? When does blood bicarbonate peak in the blood? (9) What happens when sodium bicarbonate is taken after exercise instead of before? (10) Should sodium bicarbonate be taken throughout training, or can it be taken just during the day of a competition/game/race? Are there benefits of taking sodium bicarbonate for training adaptations? (11) Which sports mainly benefit from sodium bicarbonate supplementation? (12) Should one worry about the sodium dose? (13) How does extracellular buffering compare to intracellular buffering agents such as beta-alanine? (14) Is there a possible placebo effect with sodium bicarbonate? (15) Are the ergogenic effects of sodium bicarbonate sex-specific? To address these questions, we conducted an evidence-based review of the literature on sodium bicarbonate supplementation, with contributions from several invited experts. Comprehensive answers are provided alongside suggestions for future research.

## 1. Introduction

Sodium bicarbonate (SB), commonly known as baking soda, is an extracellular buffering agent with increasing research evidence supporting its use as an ergogenic aid. It is commonly ingested in doses from 0.2 to 0.5 g·kg^−1^ body mass (BM) and intended to improve performance that utilizes non-oxidative metabolism. Its body of research in exercise performance began nearly a century ago by Dennig et al. at the Harvard Fatigue Laboratory [1]. Using a single healthy male participant, researchers induced acidosis by ingesting ammonium chloride (NH_4_Cl^−^) and alkalosis with SB. These authors measured blood pH, carbon dioxide (CO_2_) production, “lactic acid” accumulation, and work performance during treadmill running [1]. These results showed that the acidosis response associated with the exercise task significantly reduced the blood's buffering capacity and its ability to buffer the increase in hydrogen ions (H^+^) associated with lactate accumulation, resultantly limiting the runner's endurance capacity. Since this time, a position paper by the International Society of Sports Nutrition (ISSN) concluded that SB supplementation (0.2–0.5 g·kg^−1^ BM) enhances performance in high-intensity exercise lasting 30 s to 12 min, including muscular endurance and combat- or sprint-based sports. This ergogenic aid benefits both men and women in single and repeated efforts. The optimal single dose is 0.3 g·kg^−1^ BM, taken 60–180 min before exercise (depending on the ingestion form), whereas higher doses offer no added benefit and increase side effects (e.g. bloating, nausea). Multi-day loading (0.4–0.5 g·kg^−1^ BM per day for 3–7 days), divided into smaller doses, can improve performance and reduce discomfort [2].

1. What are sodium bicarbonate’s proposed mechanisms for enhancing performance?

There is robust evidence supporting the ergogenic effects of SB on exercise performance [3]; however, the potential gastrointestinal symptoms (GIS) associated with the compound likely reduces its usage amongst athletes [4]. Strategies, however, have been developed and researched to mitigate the GIS associated with the extracellular buffer [5–7]. To further address issues related to SB use during exercise, we conducted a narrative review of the literature to provide an evidence-based assessment of common misconceptions about its use, as SB supplementation strategies have been innovated since the publication of the aforementioned position stand. What are sodium bicarbonate's proposed mechanisms for enhancing performance?

The ergogenic effects of SB ingestion on high-intensity exercise performance are attributed primarily to an improved extracellular buffering capacity, which helps maintain acid‒base balance by attenuating the decline in the intracellular pH. The ingestion of SB elevates the extracellular bicarbonate ([HCO_3_^−^]) concentration, with increases of +5 mmol·L^−1^ typically associated with these ergogenic effects [8]. Since the sarcolemma is impermeable to bicarbonate, SB ingestion widens the electrochemical pH gradient between the intra- and extracellular regions [9], providing further protection against intramuscular acidosis [10,11]. This corresponding increased gradient also stimulates lactate/H^+^ co-transporter, mediated by two isoforms of monocarboxylate (MCT) transporters (MCT-1,4) [12], thereby enhancing the lactate and H^+^ efflux from contracting muscle fibers [11,13]. Given that the accumulation of H^+^ (i.e. acidosis) is considered a primary contributor to muscle fatigue during high-intensity exercise (though this remains debated) [14–16], the ergogenic effects of SB ingestion are therefore largely attributed to enhanced extracellular buffering capacity and H^+^ efflux [17]. Furthermore, maintaining the intramuscular pH following SB ingestion may preserve important metabolic processes that are impaired by H^+^ accumulation, including key glycolytic enzyme activity (i.e. phosphofructokinase), sarcoplasmic reticulum calcium (Ca^2+^) release [18,19], Ca^2+^ binding affinity to troponin [20] and the actin-myosin cross-bridge cycling [21,22]. Collectively, SB ingestion likely delays the onset of fatigue during high-intensity exercise by reducing H^+^ accumulation and preserving the intramuscular pH.

In addition to its buffering effects, SB ingestion alters the movement and distribution of strong ions (i.e. Na^+^, K^+^, Cl^−^, and Ca^2+^), consequently influencing the extracellular strong ion difference (SID), a major element of acid‒base balance homeostasis [23], which has important implications for fatigue during high-intensity exercise performance. Indeed, the changes in SID, led predominantly by increases in extracellular [Na^+^] and reductions in extracellular [Cl^−^], contribute to alkalosis (increase in blood pH and blood HCO_3_^−^) and facilitate H^+^ efflux out of the cell [24,25]. Furthermore, the intra- and extracellular equilibrium of these ions is crucial for maintaining skeletal muscle excitability and contractile function [26]. Increased extracellular [K^+^] efflux during intense muscle activity can cause membrane depolarization, consequently weakening muscle excitability and impairing tetanic force production [27,28]. The ingestion of this alkalizing agent has been shown to attenuate increases in extracellular [K^+^] during both short [29,30], intermittent [31], and prolonged [32] bouts of high-intensity exercise; however, this is not always observed [33]. Such inconsistent findings may reflect differences in exercise modality, participant characteristics, supplementation strategies, and demands of the exercise protocol. For example, Newbury et al. [33] demonstrated that SB increased [HCO_3_^−^] but did not attenuate K+ accumulation or improve performance. As such, the modulation of extracellular K+ may be context-dependent, and its contribution to the erogenicity of SB may be secondary to its established extracellular buffering capacity.

Previous work comparing SB during repeated swimming bouts did not find differences in performance or apparent SID compared to placebo, including no reduction in extracellular [K⁺] [33]. The lack of an ergogenic effect may reflect the minimal change in SID, as insufficient shifts in strong ions after ingestion may not have meaningfully influenced fatigue-related muscle excitability. Nonetheless, the corresponding reductions in extracellular [K^+^] observed from previous work demonstrated that the ergogenic effects of SB [29,30,32] also occurred concomitantly with decreases in extracellular [Cl^−^], likely suggesting that intracellular uptake to preserve excitation–contraction coupling [26]. Similarly, increased intracellular [Cl^−^] and [K^+^] uptake was observed during exhaustive finger flexion exercise; however, these authors also observed a greater [K^+^] efflux [34]. Despite this, the elevated [K^+^] efflux likely reflected the ~25% greater work output following induced alkalosis when compared to the control trial. It is probable that SB ingestion facilitates greater intracellular [K^+^] influx while limiting [K^+^] efflux into the extracellular compartment [34], helping maintain cell membrane excitability during repeated high-intensity muscle contractions [35]. Although evidence regarding the specific role of extracellular [Cl^−^] on fatigue during high-intensity exercise is limited, the interactions between [K^+^] and [Cl^−^] likely influence muscle excitability during high-intensity exercise [36]. This suggests that SB ingestion improves extracellular buffering capacity and strong-ion regulation which helps uphold membrane excitability to support this alkalizing agent's ergogenic effects on high-intensity exercise performance.

Outside of the well-established peripheral effects associated with SB ingestion, limited evidence suggests that SB-induced alkalosis may attenuate central fatigue by decreasing the rating of perceived exertion (RPE) [37–39]. Improvements in leg-RPE recovery were observed during repeated cycling bouts at 90% peak oxygen uptake (VO_2Peak_) following SB ingestion when compared to placebo, but a split-dose of SB did not reduce perceived effort, leaving its efficacy in this regard unclear [39]. However, in later work measuring central drive via voluntary activation during an integration of ischemia and peak contractions, SB ingestion preserved voluntary activation to a greater magnitude (76% ± 5%) when compared to control (57% ± 8%) [40]. Preserved voluntary activation following SB ingestion may stem from a reduction in group III and IV afferent fibers when acidosis is attenuated, as these fibers are stimulated by elevated intracellular H^+^ [41]. Therefore, this mechanism could contribute to reduced central fatigue and lower perceived effort. Similarly, recent research has demonstrated improvements in prolonged high-intensity exercise performance after SB ingestion with a reduced relative oxygen cost, likely suggesting enhanced gross economy and efficiency [32]. Nonetheless, further work is needed to clarify the potential central-related and reduced relative oxygen cost that may contribute to the ergogenic effects of SB ingestion on high-intensity exercise performance beyond its widely recognized role in H^+^ buffering. Collectively, SB ingestion likely improves exercise performance through a combination of peripheral and central mechanisms that help maintain muscle function and neural drive under high-intensity conditions.

Summary: Acute SB ingestion enhances high-intensity exercise performance primarily by increasing the extracellular buffering capacity, which subsequently promotes greater H⁺ efflux, thereby preserving the intramuscular pH and key metabolic and contractile processes. The ingestion of this ergogenic substance also influences the equilibrium of strong ions, which helps maintain muscle excitability during repeated high-intensity efforts. Beyond these peripheral effects, limited evidence suggests that SB may also reduce central fatigue by preserving voluntary activation and lowering perceived exertion.

2. Does sodium bicarbonate cause gastrointestinal symptoms, and why?

The most documented adverse side-effects from SB supplementation are GIS [2], particularly when ingested in an aqueous solution or gelatin capsules [42]. The presence of GIS may cause SB to be ergolytic in certain individuals [43], while others still show ergogenic benefits from the alkalizing agent in spite of GIS. Despite its effects on performance remaining equivocal, GIS may prevent athletes and coaches from administering SB and provide practical limitations when administering SB in multi-day sporting events.

Gastrointestinal symptoms are often prevalent when SB is ingested in solution or non-gastro-resistant capsules, as the interaction of the supplement with stomach acid contributes to the accumulation of CO_2_ in the gastrointestinal tract, thereby causing nausea, bloating, vomiting and abdominal pain [4]. Importantly, these adverse effects may be dose-dependent dose dependent [44,45], especially when ingested in solution or non-gastro-resistant capsules. Several studies have shown that SB supplementation (0.3 g·kg^−1^ BM) can interfere with exercise performance, possibly due to GIS associated with supplementation [43,46,47]. Therefore, lower doses of SB supplementation (<0.3 g·kg^−1^ BM) may serve as an alternative intervention to attenuate GIS over time if choosing such ingestion forms. Another possible strategy to help alleviate potential adverse effects associated with SB supplementation is related to the timing of ingestion. For example, Siegler et al. [48] showed that SB supplementation 3 h before repeated trials of sprint exercise in healthy young males resulted in a lower GIS compared to ingestion 1 h and 2 h before exercise. Furthermore, Carr et al. [42] showed that the greatest incidence of GIS occurred 1.5 h after supplementation with 0.3 g·kg^−1^ BM of SB, whereas the lowest incidence occurred 2 h after ingestion. In contrast, chronic SB ingestion strategies have been explored to reduce the GIS response, particularly in wrestling [49]. Similarly, progressive chronic dose strategies over a 10-day period prior to exercise have been employed [50]. Although this research is scarce, ergogenic effects in Cross-Fit-like performance have been observed without any significant differences in GIS when compared to placebo [50]. Importantly, without a trial comparing this SB ingestion strategy to typically employed acute SB ingestion strategies, it is not possible to elucidate the superiority of the progressive dose strategy and instead provides a future avenue for further research.

In addition to the timing of ingestion, there is evidence to suggest that the delivery method of SB may help reduce the incidence of adverse effects. Although based on a small sample of research, there is evidence that the co-ingestion of SB with a high-carbohydrate meal (1.5 g·kg^−1^ BM) induces alkalosis while also reducing GIS [42]. Additionally, while issues are predominantly observed following the ingestion of SB in forms that are susceptible to interaction with stomach acid, consuming SB in enteric-coated capsules or delayed release capsules appears to minimize the incidence of GIS [5]. Such capsules are encapsulated in an enteric coating, which is designed to reduce interaction with stomach acid [5]. Finally, the Maurten Bicarb System (SBH) (Maurten AB, Gothenburg, Sweden) has been designed to minimize GIS through a novel delivery system, delivering SB mini-tablets in a patented carbohydrate (CHO) hydrogel [7,51]. Briefly, the ingestion form may have a large impact on subsequent GIS; however, SB's various forms are more thoroughly discussed in question #3.

Summary: Taken together, there is evidence that SB supplementation in some forms can result in adverse effects, primarily involving GIS. These effects are more evident at a dose of 0.3 g·kg^−1^BM (or higher) as well as in non-gastro-resistant formulations. Alterations in the relative dose, timing of ingestion, and delivery method of SB supplementation should be considered to alleviate possible GIS-related side effects. Additionally, athletes should experiment with different forms and ingestion strategies in practice to avoid a GIS in competition. Athletes and coaches should be mindful that competition itself may also cause some GIS, so they should employ the SB ingestion strategy, which provides the lowest possible GIS in training, in the knowledge that GIS may be more severe in competition.

3. What are the different ways in which sodium bicarbonate can be ingested and how does this affect blood bicarbonate changes, and exercise performance?

Sodium bicarbonate is a well-established extracellular buffering agent; however, its effectiveness and practicality may depend on its form of ingestion. For example, SB can be ingested in aqueous solution, non-gastro-resistant capsules (vegetarian or gelatin capsule), gastro-resistant capsules (delayed release and enteric coated capsules) or newer, commercially available hydrogel delivery systems (e.g. SBH). Each form may subsequently affect blood bicarbonate changes, side effects, and performance enhancement.

Sodium bicarbonate has most commonly been ingested when dissolved in water or in numerous gelatin capsules [3]. In both cases, SB is released into the stomach within minutes, as conventional hard gelatin capsules disintegrate rapidly under gastric conditions [52]. Nonetheless, there appear to be differences in the pharmacokinetics of circulating bicarbonate between these ingestion methods. For example, the mean individual time-to-peak (ITTP) blood bicarbonate concentration is likely to emerge faster (≤100 min) following the ingestion of 0.3 g·kg^−1^ BM in solution [29,53,54] when compared to ingesting SB in size 00 gelatin capsules (>100 min) [55,56]. Still, there is a large inter-individual component with both forms [53,56] and even capsule size might also modify this response [57]. Interestingly, while ITTP has been shown to be consistent following SB consumption via solution [53], the same has not been shown for capsules [55]. Nonetheless, the need for ITTP strategies has been questioned [3,55]. Importantly, it has been proposed that a 5 mmol·L^−1^ increase is necessary to improve performance, particularly when ingesting SB in solution or in gelatin capsules. Consequently, blood bicarbonate increases of >5 mmol·L^−1^ are commonly achieved ~60 min after the ingestion of SB in solution [53,56] and 75 min after the ingestion of SB in non-gastro-resistant capsules [55] and remain elevated above these thresholds for several hours, suggesting a prolonged ergogenic window of opportunity with both of these forms.

Novel encapsulated ingestion forms designed to minimize GIS-related side-effects include delayed release [58] and enteric coated [59] capsules. In previous research, delayed release capsules slowed down the onset of blood bicarbonate increases (32 vs. 20 min) and ITTP (120 vs. 72 min) compared to a solution [58], without the observation of any absolute change in blood bicarbonate. Similarly, enteric capsules delayed the ITTP blood bicarbonate response when compared to ingestion of SB in gelatin capsules (110 vs. 90 min), without any differences in peak blood bicarbonate concentrations [59]. A mini-tablet formulation that is ingested with a patented CHO hydrogel system (SBH) helps deliver mini-tablets directly through the pyloric sphincter because of their small size, avoiding interaction with stomach acid [7]. A time course study comparing this formulation to the ingestion of SB in vegetarian capsules (size 00) showed modestly greater overall blood bicarbonate responses (+0.95 mmol·L^−1^) and faster ITTP increases (42.5 min) with this novel system. Nonetheless, while small differences may exist, all available SB forms increase the blood bicarbonate concentration sufficiently over several hours to enhance exercise performance, particularly if the GIS is not experienced.

Irrespective of the ingestion form, SB appears to improve exercise performance, as evidenced by several systematic reviews and meta-analyses [3,42,60,61]. Individual studies have shown performance benefits following ingestion in solution [45,62–65], gelatin capsules [10,37,66–69], delayed release or enteric capsules [58,59,70] and SBH [32,51]. A meta-analysis showed ingestion in solution leads to modestly greater performance benefits than capsules [3], though these data combined all the extracellular buffers (SB, sodium citrate and sodium/potassium lactate). Since dissolving SB in solution may lead to impaired blinding [71], placebo effects may account for the greater benefits seen with solution [72] in studies that did not sodium match their placebo. Traditional intake methods (e.g. solution or standard capsules) may be more suited to acute, high-intensity exercise bouts, whereas formulations with comparatively longer pharmacokinetic curves (e.g. delayed release or SBH) could also provide sustained elevations in blood bicarbonate and thereby confer greater benefits during prolonged exercise. It is unclear if the variability in exercise responses to SB [43,47] is due to the ingestion form (capsules) or a true intra-individual response. Avoiding side-effects may be key in optimizing the beneficial effects of SB [47,73]; however, all forms of SB have the potential to improve performance.

Summary: While ingestion form may lead to some differences in the blood bicarbonate pharmacokinetics, each form of SB can induce alkalosis to a degree deemed necessary to improve exercise performance (e.g. 5 mmol·L^−1^ increase in blood bicarbonate). Regardless of the ingestion form, SB ingestion improves high-intensity exercise performance; however, forms of SB that interact with stomach acid (i.e. solution, gelatin capsules) are more susceptible to GIS. Therefore, selecting a form of SB that reduces the interaction with stomach acid will enhance practicality and will likely optimize performance to a greater degree.

4. How does sodium bicarbonate affect performance in short-duration exercise (≤10 min)?

Athletes, sport practitioners, and coaches often implement SB supplementation prior to high-intensity exercise. Although discussed in more detail in Question 1, SB ingestion is more likely to benefit high-intensity exercise, which is more reliant on non-oxidative metabolism. Such exercise produces large quantities of lactate and protons in the intracellular compartment, facilitating greater efflux of these ions, which supports extracellular H^+^ buffering [17]. Additionally, during high-intensity exercise, extracellular K⁺ increases contribute to the impairment of muscle excitability. However, SB ingestion can limit this strong ion's efflux by preserving this electrolyte's influx in the intracellular compartment, and instead, aid in the protection of muscle excitability [26]. Since SB ingestion exerts its ergogenic effects by enhancing extracellular buffering under conditions of large H⁺ accumulation and extracellular K⁺, supplementation with this extracellular buffer benefits high-intensity exercise performed within 30 s to 10 min. Consequently, SB ingestion has since demonstrated performance improvements corresponding to this range, including 4 km cycling time trial (TT) performance [59,62], 2 km rowing performance [74], and 400 m [75] and 1500 m [46] running performance. Despite these positive findings, which demonstrate the ergogenic effects of SB ingestion for high-intensity exercise performance up to 10 min in duration, this is not always observed [69,73,76]. In some cases, the lack of an improvement in high-intensity performance may be attributed to ingestion strategies that failed to coincide with peak alkalotic concentrations, thereby limiting the ergogenic potential of the extracellular buffering agent [77]. Other reasons underpinning the lack of a performance benefit in some studies may be attributed to the variability in ingestion strategies that may have induced GIS, potentially even resulting in ergolytic performance responses [4,43,73].

It is possible that the ergogenic effects of SB on high-intensity exercise lasting between 30 s and 1 min in duration are smaller, given the limitations imposed by its mechanism of action. For example, supramaximal exercise lasting approximately 30 s is associated with a rapid and pronounced decline in intramuscular pH, which can saturate MCTs with H⁺. This corresponding saturation further limits proton efflux and likely nullifies the beneficial effects of increased circulating bicarbonate, arguably reducing the capacity for SB ingestion to exert ergogenic benefits [22]. This may help elucidate why meta-analytical data did not find an ergogenic effect of SB ingestion compared with placebo on 30 s high-intensity cycling performance (30 s Wingate test), with a trivial standardized mean difference (SMD = 0.02) [78]. Nonetheless, previous research has demonstrated ergogenic effects of SB ingestion within the 30 s to 1 min duration in exercise such as standalone and repeated 30 s cycling bouts (e.g. Wingate test) [79,80], 2 × 100 m swimming bouts [81], and 4 × 50 m swimming bouts (4 × 30 s) [82]; however, this is not always the case [64,83–85]. Collectively, these findings suggest that while SB ingestion may generate ergogenic benefits during some high-intensity exercise bouts lasting 30 s to 1 min, these effects are less consistent and generally smaller compared with longer-duration efforts, likely because of mechanistic constraints on proton efflux and extracellular buffering.

Summary: sodium bicarbonate ingestion is renowned for improving high-intensity exercise performance up to 10 min in duration. This contrasts to high intensity exercise up to 30 s in duration, whereby the rapid decline in the intracellular pH inhibits sufficient extracellular buffering since the MCT transporters become saturated with H⁺ and subsequently diminishes the ergogenic capacity of SB ingestion. Therefore, SB ingestion enhances high-intensity exercise performance up to 10 min in duration, with reduced benefits during very short bouts of exercise (<30 s) owing to an attenuation in proton buffering.

5. How does sodium bicarbonate affect performance in long-duration exercise, and should it be used before and during such events?

Historically, the ergogenic benefits of SB ingestion have been associated with short (<10 min) high-intensity exercise, with most research focusing on exercise of this duration, as evidenced in a recent meta-analysis [3]. Despite extensive research examining SB in short high-intensity bouts, widespread anecdotal reports also suggest that its benefits extend to long-duration events such as Grand Tour cycling stages and trail ultra-running events. Indeed, case report data may support its use in long-duration events, given that blood lactate concentrations in an elite ultra-endurance athlete have been measured as high as 19 mmol·L^−1^ late in an ultramarathon event [86], an acid‒base stress that may benefit from an extracellular buffer. Indeed, as discussed in question 1, one of the primary mechanisms for SB's ergogenic effects is its ability to buffer disproportionate H^+^. Although short-duration exercise is typically associated with the accumulation of this intramuscular metabolite, this does not eliminate prolonged high-intensity exercise as a condition by which disturbances in acid‒base balance may also contribute to exercise-induced fatigue and performance impairment. Importantly, the degree by which H^+^ accumulates is largely dependent on exercise intensity [87], with greater perturbations in acid‒base balance occurring when exercise is performed at or above heavy and severe domain intensities. Accordingly, exercise performed within the heavy-domain may be sustained for prolonged durations (up to 3–4 h) [87], whereas exercise above critical power (i.e. severe-domain) can usually be sustained for several minutes (~2–15 min) [88]. Given the competitive demands of outperforming residual competitors, particularly during end-spurts or final sprints, athletes are therefore more than likely to perform within heavy- and severe-intensity domains, where these athletes may benefit from enhanced extracellular buffering capacity. Consequently, there is increasing evidence to support SB's efficacy on prolonged high-intensity exercise performance [6,32,70,89–91]; however, this is not always observed [92–94]. These contrasting findings may, however, be attributed to differences in experimental design, especially regarding the variety of ingestion strategies that have been employed.

Stephens et al. [94] did not find a performance benefit when SB was ingested in gelatin capsules 120 min before 30 min exercise bout at ~80% VO_2Peak,_ likely missing peak alkalosis (60–90 min) [42,59], and hence diminishing the potential of an ergogenic effect. McNaughton et al. [91] and Egger et al. [90] observed improvements in 1 h TT performance and cycling at 110% of the individual anaerobic threshold (IAT) following the ingestion of SB in aqueous solution, which elevates blood bicarbonate at a quicker rate when compared to capsules [58], likely aligning the start of exercise (60 min post-ingestion) closer to peak alkalosis. This is similar to recent work, where researchers have made concerted attempts to align exercise with ITTP alkalosis following ingestion, which has likely assisted in improving prolonged bouts such as 3.5 km TT running performance [95] and 16.1 km [70] and 40 km [32] cycling TT performance. It is therefore possible that the timing of SB ingestion has important implications for aligning peak alkalosis with exercise onset [77], which may be important for maximizing its ergogenic effects for prolonged high-intensity exercise. However, without a study design that directly compares a standardized ingestion strategy vs. an ITTP ingestion strategy on prolonged high-intensity exercise performance, it is not possible to elucidate whether one is superior. Conversely, previous research has suggested that ITTP strategies may not be necessary given that, in gelatin capsules, Bayesian modeling indicates that more than 80% of the sample population will achieve a 5 mmol⋅L^−1^ increase in blood bicarbonate between 75 and 240 min after SB ingestion [55]. Given this wide timeframe by which participants will achieve a meaningful increase in blood bicarbonate after the ingestion of SB in traditional capsules, the practical necessity of precisely individualizing ingestion timing using this form of SB could be reduced.

This is comparable to recent research investigating a novel commercially available ingestion system (SBH) [51], in which all participants (*n* = 10) achieved a 5 mmol·L^−1^ increase in blood bicarbonate by 90 min. Taken together, it is possible that these data suggest that an ITTP ingestion strategy is not necessary with some supplementation forms, which is, however, contingent upon the assumption that the magnitude of the observed increase in blood bicarbonate is directly associated with the ergogenic potential of SB ingestion. Therefore, the aforementioned improvements in prolonged high-intensity exercise performance after SB ingestion using these forms [32,70] may be less attributed to the optimization of peak alkalosis, given that it is probable that standardized timing protocols may be sufficient in inducing alkalosis to thresholds associated with the ergogenic effects of SB ingestion. Nonetheless, mixed findings in prolonged high-intensity exercise performance after SB ingestion may be attributed to laboratory-based exercise protocol or differences in supplementation strategies used.

Exercise protocol type may further play a role in whether SB will improve long-duration exercise performance. For example, the protocols showing benefits from SB supplementation [32,70,95] utilized ecologically valid TT protocols, which better represent races than time-to-exhaustion (TTE) protocols and demonstrate a lower coefficient of variation [96]. Mechanistically, TT protocols may better reveal SB's ergogenic effects, as TTs involve intensity fluctuations that increase reliance on extracellular buffering. Indeed, varying pacing has been shown to produce greater muscular fatigue, blood lactate, heart rate, ventilation, and oxygen consumption when compared to a steady, workload-matched bout [97,98]. Previous investigations [93,99] did not report an improvement; for example, following SB ingestion, the extracellular buffer did not improve TTE at 110% IAT after running at 95% IAT for 30 min. However, these findings differ to those by Egger et al. [90], where a ~4.5 min improvement in TTE was observed following SB ingestion compared to placebo. Future research should therefore aim to clarify these contrasting effects while utilizing prolonged, high intensity, and ecologically valid protocols.

Gastrointestinal symptoms warrant consideration when administering SB for prolonged high-intensity exercise, as such exercise may be more susceptible to GIS in comparison to short duration exercise. Furthermore, the variability of GIS responses may contribute to inconsistent performance outcomes [93]. Previous long-duration SB research has also implemented various ingestion strategies to mitigate GIS, as Dalle et al. [6] found a performance improvement accompanied by minimal GIS in sprint performance during a 3-h simulated cycling race after ingesting SB (0.3 g·kg^−1^ BM) with a “split-dose” protocol (i.e. over the course of several hours). Additionally, using gastro-resistant forms of SB, such as delayed-release and enteric-coated capsules has led to observations of improvements in prolonged running [95] and cycling performance [70]. These encapsulation methods, however, require the inconvenient ingestion of up to 20–25 capsules for a 70–75 kg athlete, potentially rendering this ingestion method impractical. Alternatives such as SBH have demonstrated reductions in GIS in comparison to encapsulated SB at rest [7] while enhancing short [51] and prolonged [32] high-intensity cycling performance. Nonetheless, little research exists directly comparing the effects of SBH with other SB formulations on exercise performance, limiting conclusions regarding the potential superiority of any specific delivery method. Taken together, SB ingestion likely enhances prolonged high-intensity exercise performance; however, its effectiveness may depend on timing, protocol design, and ingestion form. Overall, this alkalizing agent may be a valuable ergogenic aid for long-duration high-intensity exercise when both physiological and logistical considerations are addressed and optimized.

Summary: Sodium bicarbonate has predominantly been examined in short-duration high-intensity exercise, yet emerging evidence suggests that it may also improve prolonged high-intensity performance. These mixed findings likely reflect differences in the timing of ingestion, ingestion form, GIS, and exercise protocol. However, when these factors are optimized, SB appears to be a viable ergogenic aid for long-duration high-intensity exercise.

6. Is there a potential rationale for sodium bicarbonate use at altitude?

Whilst the ergogenic effects of SB ingestion are well-established at sea level, its benefits are less understood in extreme environments, such as altitude. Given that many major international competitions, such as World Championships and the Olympic Games, are frequently hosted in locations involving exposure to altitude, it is paramount to understand if SB ingestion can provide ergogenic effects under this stressor. A recent narrative review [100] assessed the effects of buffering agents at altitude on exercise performance, including sodium citrate (*n* = 3), beta-alanine (*n* = 2), and sodium bicarbonate (SB; *n* = 8). Across these included studies conducted under hypoxic conditions (*n* = 13; mean ± standard deviation simulated elevation = 3181 ± 1024 m), only 23% reported ergogenic effects. These findings highlight the necessity of elucidating the physiological mechanistic rationale for SB ingestion at altitude before its ergogenic potential in this condition can be fully understood.

Previous research has demonstrated performance improvements in acute normobaric hypoxia following SB ingestion [29,101–104]; however, this is not always observed [105,106]. The improvements in exercise performance following SB ingestion in acute normobaric hypoxia have predominantly been observed for short high-intensity exercise [101,102,106], although recent evidence suggests that these ergogenic benefits extend to prolonged high-intensity exercise performance [103]. Additionally, apart from Saunders et al. [105] and Shannon et al. [103], each of the studies investigating SB ingestion in acute normobaric hypoxia has adopted a simulated altitude of ~3000 m, which is a height that is unlikely to be experienced by athletes since most training camps and competition venues are situated <~2000 m [100]. Nonetheless, the improvements in performance after SB ingestion are likely attributed to an enhanced extracellular buffering capacity pre-exercise, given the exacerbated accumulation of H^+^ in this environment and the subsequent need for extracellular buffering sources [107]. Conversely, acute sojourns to altitude result in respiratory alkalosis due to hyperventilation associated with increased ventilatory drive caused by a lower partial pressure of oxygen (PO_2_). Over the course of hours to days, the kidneys decrease the rate of bicarbonate reabsorption to return pH back to normal (in the process of lowering baseline levels of bicarbonate) [108]. The extent and effect of these acid‒base adjustments would most likely vary across individual athletes and altitudes, which therefore requires serial measurements to better track changes in blood buffering across training or competition cycles at various heights. This may also explain the disparate findings in the literature regarding prolonged exposure and SB supplementation [109,110]. Conversely, SB supplementation early in the exposure period and during acute repeated, high-intensity training may provide athletes with supplemental bicarbonate to overcome any existing blood buffering deficit [101,106,111], which may, at least partially explain the improvements in high-intensity exercise performance. Ultimately, it is still unclear whether the use of SB supplementation (acutely or chronically) at altitude will improve performance or training adaptations in athletes, warranting further research in this area.

Summary: There is a physiological rationale for the use of SB at altitude. It is likely that SB ingestion at altitude will provide supplemental bicarbonate to alleviate the potential reduction in blood bicarbonate commonly observed at altitude; however, its effects on exercise performance and training adaptations in this environment are less clear. Similarly, most of the research assessing SB's ergogenic potential at altitude has occurred at heights less likely to be experienced by athletes, and more research elucidating SB's effectiveness at externally valid heights is required.

7. Is it worth ingesting sodium bicarbonate for exercise in the heat?

Like sea level, the effects of SB are well-established in temperate environmental conditions; however, there have been few concerted efforts to investigate this extracellular buffer's ergogenic potential in heat. Accordingly, Carr et al. [100] demonstrated in a recent narrative review that only five investigations (sodium citrate, *n* = 3; SB, *n* = 2) studied the effects of extracellular buffering supplementation in heat (mean ± standard deviation temperature = 31.4 °C ± 2.2 °C), and only one of these included studies observed ergogenic effects. Despite the lack of research and observed ergogenic effects of SB on exercise performance in heat, SB ingestion may serve additional purposes outside of improving exercise.

Recently, the use of SB supplementation for the purpose of either hyperhydrating or expanding plasma volume to mitigate the effects of heat stress during prolonged exercise has been explored [14]. Interestingly, the amount of sodium (Na^+^) ingested with a typical dose of SB is nearly equivalent to Na^+^ levels used during salt-only hyperhydration studies [112,113], and the resulting plasma volume expansion is similar (~ 6%–8%) [14]. However, whether this expansion benefits cardiovascular performance and/or thermoregulation during exercise in heat remains unclear. Preliminary evidence suggests performance benefits under heat stress, with Mündel et al. [80] reporting improved recovery and repeated anaerobic capacity during Wingate testing in the heat following SB ingestion, and Gough et al. [114] demonstrating enhanced 4 km cycling TT performance using an ITTP ingestion strategy in hot conditions (30 °C). Supplementary to these performance-related studies, while investigating cycling in heat (35 °C) for 60 min at 50% VO_2Peak_, Katagiri et al. [115] demonstrated reduced minute ventilation in the SB condition (57.1 ± 9.1 L·min^−1^) when compared to placebo (62.1 ± 9.1 L·min^−1^) at the end of exercise. Similarly, these authors also observed significantly reduced RPE after the ingestion of SB (15.6 ± 1.8 AU) in comparison to placebo (16.0 ± 1.8 AU), suggesting that this extracellular buffer reduces heat-induced hyperventilation. More work has been done with both SB and sodium citrate in the area of rehydration after rapid body mass loss (e.g. making weight in wrestling or combat sports), with both agents demonstrating greater efficacy in increasing plasma volume, body mass, and water consumption when compared to water-only controls [116–118]. Another potential use of SB supplementation during heat stress is to reduce the risk of acute kidney injury by potentially preserving acid‒base balance and lowering the kidney's energy requirements for bicarbonate reabsorption, reducing the damage caused by the breakdown of muscle tissue (e.g. rhabdomyolysis), or improving renal function directly via the high sodium content [119]. In this context, SB supplementation may be considered for both athletes competing for prolonged periods in heat (e.g. marathon or ultras) or occupations where recurring prolonged heat exposures persist (e.g. construction, firefighters, etc.).

Summary: Although research investigating SB in hot conditions is limited and its ergogenic effects in this environment are limited, this extracellular buffer's purpose may serve beyond just for exercise performance. Given its heightened sodium content, SB may promote plasma-volume expansion and hyperhydration when compared to traditional salt-loading strategies, which may subsequently support cardiovascular function and thermoregulation during heat stress. Preliminary work also demonstrated improvements in high-intensity exercise performance, reduced perception of effort, and lower ventilation. Overall, further research is necessary to elucidate the efficacy of SB ingestion for improving hydration status, cardiovascular and thermoregulatory function, exercise performance, and renal function during prolonged exercise and occupational heat exposure in hot environments.

8. When is the best time to take sodium bicarbonate before exercise? When does blood bicarbonate peak in the blood?

Since the work of Dennig et al. [1] using split-day fluid SB ingestion, a considerable number of ingestion methods and protocols have been used to deliver SB prior to exercise. Typically, either an acute single bolus ingestion prior to exercise, or “split” dosing strategies are employed. The rationale for splitting doses, either across multiple days [120] or into smaller doses across the pre-exercise period [121], was first used to offset or limit some of the GIS that can impact some participants when SB is ingested in a single large bolus. This was particularly relevant when traditional SB ingestion forms such as fluid and gelatin capsules were the typical methods of delivery [122]. Since then, a variety of strategies have been employed (and developed) to limit the GIS (further discussed in question #2) by reducing or eliminating the interaction of SB with gastric acid [51,59] subsequently enabling large single volumes of SB to be ingested and tolerated. This diversification in ingestion type also brings specific considerations for nutrition practitioners and athletes, since each has different impacts on the subsequent blood bicarbonate response and potentially the optimal timing of intake. Such considerations are also further compounded by the variety of ingestion doses, timings of ingestion (Table 1), and individual responses that have been reported in the literature, and indeed, which athletes often experiment with. From a practitioner's perspective, considerable confusion exists among athletes and coaches about the difference between ingestion forms of SB and the specific pre-exercise ingestion considerations for each.

**Table 1.** Pre-exercise ingestion time recommendations and considerations for a variety of sodium bicarbonate (SB) intake forms.

	SB in solution	Gelatin & vegetarian capsules	Enteric coated capsules	Delayed release capsules	SBH
Ingestion time (min)	60–90	70–100	120–140	110–130	90–120
Benefit of using ITTP	Yes	Likely	Possibly	Possibly	Possibly
Likelihood of GIS^a^	High	High	Low/moderate	Low/moderate	Low
Likely severity of GIS	High	High	Moderate^b^	Moderate^b^	Low

^a^ Likelihood is based on those that are sensitive to or have a history of gastrointestinal symptoms (GIS) following SB ingestion.

^b^ Based on anecdotal reporting from participants and athletes several hours after exercise. SBH = sodium bicarbonate minitablets in patented carbohydrate hydrogel (Maurten Bicarb System). ITTP = Individual time to peak.

An acute single SB dose of 0.3 g·kg^−1^ is most widely used in the literature, as it was identified as “optimal” in a dose–response study by McNaughton [45]. However, since then, Jones et al. [56] demonstrated that there is considerable inter-individual variability in the blood bicarbonate response across doses and individuals, making it difficult to recommend an optimal pre-exercise ingestion time for SB. Research on this individual variability concept was further developed by both Miller et al. [54] and Sparks et al. [123] with the establishment of the ITTP alkalosis ingestion strategy. The premise of this strategy was to determine when alkalosis peaks (via blood pH) following SB ingestion, with the assumption that the largest increase in blood pH may provide the greatest likelihood of an ergogenic effect [12]. Subsequent work by Gough et al. [62], however, observed that measuring peak blood bicarbonate was a more reliable method than measuring peak blood pH, and later showed that adopting a strategy based on ITTP blood bicarbonate improved 4 km cycling TT performance, whereas this was not the case for standardized ingestion timing [124,125]. Indeed, direct comparison of an ITTP ingestion to standardized ingestion time was shown by Boegman et al. [77] to provide an advantage; however, the standardized ingestion time of 60 min adopted in this study was unlikely to induce alkalosis to a sufficient degree in comparison to other standardized times, such as 90 min. Similarly, other instances observing ITTP alkalosis may be less reliable when ingesting SB using gelatin (or similar) capsules. This is likely the result of capsule aggregation in the stomach, potentially reducing the ergogenic effect of individual timing [55] and impacting bicarbonate absorption rates, which can also be affected by capsule size [57]. Whilst the ITTP strategy may provide a performance advantage in some instances, it is time consuming and requires access to a blood gas analyzer, limiting its practicality for some athletes.

**Figure 1.**
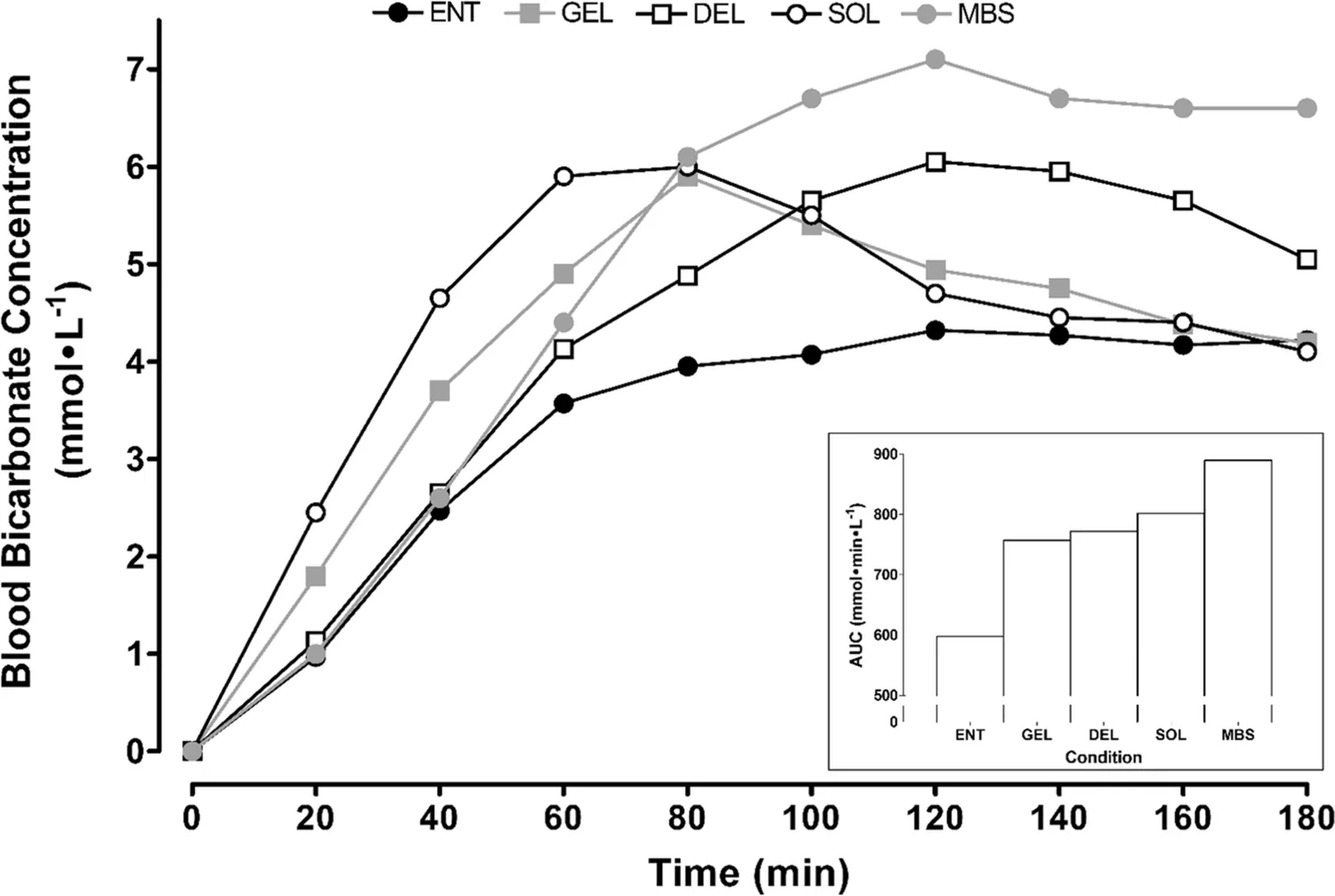
Mean change (D) in blood bicarbonate concentrations over 3 h at rest following ingestion of 0.3 g·kg^−1^ sodium bicarbonate and area under the blood bicarbonate curve in a variety of frequently used ingestion types. ENT = enteric coated capsules, GEL = gelatin capsules, DEL = delayed release capsules, SOL = solution, SBH = sodium bicarbonate mini-tablets in patented carbohydrate hydrogel (Maurten Bicarb System). Data redrawn from Hilton et al. [59] and Gough and Sparks [7].

Employing gastro-resistant capsules or tablets to reduce the GIS may allow more bicarbonate to be delivered, whilst lowering the peak and lengthening the time after ingestion such that the highest blood bicarbonate response is observed [5]. This may however also allow blood bicarbonate to be elevated for considerably longer than with a fluid ingestion strategy, so while the 3 h post ingestion area under the blood bicarbonate curve is smaller with enteric coated capsules (Figure 1), it is likely larger over >5 h after ingestion and may therefore be more suitable for longer duration exercise [70]. More recently, a SB ingestion system using mini-tablets and a CHO hydrogel (SBH) has been shown to elevate blood bicarbonate considerably greater than a traditional capsule form and for prolonged periods, whilst almost eliminating GIS [7,51]. The use of ITTP with this form of SB [32] has been shown to have less inter-individual variability and similar performance improvement effects when compared to a standardized ingestion time of 90 min [103]. This suggests that for this method of SB ingestion, the ITTP is likely to provide a smaller additional ergogenic effect for traditional SB ingestion forms. For the application of SB in prolonged exercise, where there is a repeated need for high intensity efforts, or when the main exertion in the race may occur at the end of a 3–5 h bout, it is important to use an ingestion form that can achieve this. Therefore, some individual assessment of when to ingest may be beneficial, and this should also consider event tactics, dynamics, and topography, as this could impact when the initial peak in blood bicarbonate might be needed.

Some endurance and ultra-endurance athletes are also experimenting with in-race “top-up” doses of SB in a variety of forms. There is anecdotal evidence of the ingestion of SB using liquid, capsule, SBH, and commercially available gels, being used to deliver a variety of in-race doses of SB. While the inspiration for such a strategy is likely the work of Dalle et al. [6], who used three split doses of SB during a 3 h cycle race simulation, this dosing strategy has not yet been evidenced in the literature as being more ergogenic than just a single optimal pre-exercise dose, and if it is useful, it is likely to be so for those SB ingestion forms that are not gastro-resistant or gastro-evasive, as these deliver less total SB (Figure 1) and the recovery of blood bicarbonate is less effective [7].

Summary: the diversification of existing SB ingestion modes produces contrasting peak blood bicarbonate responses, thereby necessitating different recommended pre-exercise ingestion times. Consequently, it is possible that the need to individualize pre-exercise SB consumption may be limited to SB forms that demonstrate large inter-individual variability in peak blood bicarbonate responses, whereas standardized pre-exercise ingestion timing strategies may be sufficient for other forms, such as SB mini-tablets in a CHO hydrogel. These findings provide valuable information for sport practitioners, nutritionists, and athletes considering SB ingestion as a pre-exercise ergogenic aid.

9. What happens when taking sodium bicarbonate after exercise instead of before?

While the majority of scientific work has focused on the efficacy of SB as a pre-exercise buffering aid, there is also scope for ingesting it after an initial exercise bout to improve recovery [126]. Rather than exogeneous bicarbonate being utilized during exercise, this approach aims to maximize one's extracellular capacity to buffer H^+^ throughout the subsequent recovery period and accelerate the restoration of blood acid‒base balance back to baseline levels or beyond [127,128]. This would be of practical significance for any athletes performing multiple events on the same competition day, where accelerated early-phase recovery is beneficial for performance. For instance, BMX cycling events involve repeated qualification rounds separated by as little as 15 min, or swimming medal competitions comprised of heats and finals, or races in different stroke disciplines, within ~30–90 min of each other. For example, at the Paris 2024 Olympics, some swimmers had as little as 82 min between the men's 200 m breaststroke and 200 m butterfly semifinals, leaving minimal time to recover between events. Furthermore, rationale exists as to why SB could also improve longer-phase recovery, ranging from a few hours up to several days post-exercise. Mechanistic studies have shown that pre-exercise SB ingestion attenuates cellular stress (e.g. intracellular heat shock protein-72, [HSP72]) [129–131] and augments mitochondrial biogenesis via the expression of peroxisome proliferator-activated receptor-γ coactivator 1-alpha (PGC-1α) [132] during recovery from strenuous exercise, which can likely be explained by enhanced H^+^ efflux from the muscle [126]. Consuming SB following the initial exercise bout instead could therefore alleviate cellular stress and promote cell repair from follow-up exercise performed on the same day, in turn accelerating recovery time and reducing the decline in functional capacity for athletes. Future studies are however warranted to further examine the effectiveness of post-exercise SB in optimizing recovery and elucidating the involved mechanisms.

A small body of work exists examining the effects of post-exercise SB ingestion through the medium of traditional ingestion modes (e.g. aqueous solution and capsules) on the restoration of blood acid-base balance and subsequent exercise performance after a short recovery [30,127,128,133]. In two similarly designed studies, Gough et al. [30,127] observed that SB consumed acutely following an initial exhaustive bout increased bicarbonate and blood pH (~7 mmol·L^−1^, ~0.10 pH units higher) 75–90 min post-exercise compared to a placebo. These elevations translated to greater exercise capacity for SB during follow-up TTE protocols (~20%). Gurton et al. [128] did not find benefits of post-exercise SB supplementation when repeated exhaustive treadmill tests were separated by 40 min, with GIS more severe throughout the subsequent 24 h. Acute SB ingestion after exercise appears more effective when sufficient recovery is available (e.g. ~60–75 min), otherwise a pre-exercise strategy should be favored to diminish the initial disturbances in the blood acid-base balance that impair subsequent performance [134]. Dalle et al. [133]found that split-dose SB supplementation across a 9 h period after repeated “all-out” cycling efforts separated by 3 h improved performance (mean power was 1.9% higher) and led to gradual increases in bicarbonate (7.6 mmol·L^−1^ greater at the end point, *p* < 0.05) compared to a placebo, without any significant differences in GIS. A split-dose post-exercise SB ingestion strategy could therefore be particularly beneficial for athletes competing in sports comprising multiple qualification rounds on a single day who are concerned about GIS impairing repeated bout performance.

Research investigating the application of post-exercise SB ingestion for accelerating recovery up to a few days after an initial exercise bout is scarce. This may be especially relevant for athletes competing in prolonged competitive scenarios (e.g. multi-week tournaments) where optimizing recovery is prioritized over long-term physiological adaptations. Gurton et al. [135] reported that SB ingested after a 40 × 15 m repeated sprint protocol (aimed to induce muscle damage) did not attenuate reductions in vertical jump height or alleviate muscle soreness through a 3-day recovery period. These authors did, however, observe that a second dose of SB consumed before a follow-up soccer-specific testing battery (~72 h post-exercise-induced muscle damage) attenuated the drop-off in Illinois Agility (4.0% less) and improved Yo-Yo IR2 (24.0% greater) performance compared to a placebo. While performance benefits may be advantageous during competitive periods, there is also a rationale that supplementation could attenuate post-exercise metabolic disturbances that contribute to physiological adaptations. Consequently, SB ingestion during off-season training blocks may be counterproductive, as the primary goal is long-term physiological development rather than minimizing small transient impairments in performance [126]. Other studies exploring the effectiveness of SB for accelerating functional and biochemical indices of recovery after eccentric or metabolic challenging exercise across a 24 h period have shown equivocal results [129–132,136]. Additional work examining the influence of post-exercise SB ingestion on cellular and adaptive responses (e.g. HSP72, PGC-1α). The application of post-exercise SB ingestion should be considered on a case-by-case basis, as accelerated recovery is most likely to be beneficial in acute performance settings where small decrements in performance between closely scheduled events or matches may determine competitive outcomes.

Summary: post‑exercise SB supplementation may enhance recovery by accelerating blood acid‑base restoration, reducing cellular stress, and improving subsequent performance, particularly when sufficient recovery time is available or with stacked dosing strategies. However, evidence remains limited and mixed, with benefits more apparent in short‑term repeated events than in prolonged recovery, while concerns exist around impeding long-term physiological adaptations. This highlights the need for further research on its physiological and practical applications, especially when adopting SB delivery forms designed to reduce GIS.

10. Should sodium bicarbonate be taken throughout training or can it be taken just during the day of a competition/game/race? Are there benefits of taking sodium bicarbonate for training adaptations?

Conceptually, like caffeine, SB provides near-immediate performance benefits, unlike supplements such as beta-alanine that may require weeks of use to be ergogenic [137,138]. Thus, from a mechanistic standpoint, SB may enhance performance even if it is consumed only on the day of the race or competition. While athletes may experience an ergogenic effect even if SB is first introduced on the day of the event without prior experimentation, they also risk impaired performance. Impaired performance may result from GIS caused by SB, particularly when ingested in liquid or simple capsule form, and may include bloating, nausea, vomiting, and abdominal pain due to the accumulation of CO_2_ in the gut [4]. For some individuals, these side effects may outweigh the performance benefits, leading to a net neutral or even negative effect. In one study including 12 athletes, SB supplementation improved 1500 m run performance in all but two participants [46]. Both participants reported abdominal discomfort and diarrhea, which potentially contributed to their non-responsiveness. Similarly, another study reported an ergogenic effect of SB only after excluding participants (4 out of 21) who experienced GIS from the analysis [43]. Therefore, athletes are strongly advised to test SB supplementation during training sessions. Testing SB in training may help: (a) identify ideal supplementation protocols; (b) become accustomed to supplementation in the case of side effects; and (c) increase the chances of realizing performance benefits on competition day.

Beyond acute performance enhancement, an important consideration is whether SB ingestion before every intense exercise session also enhances training adaptations. The current evidence is mixed, but points toward a possible benefit. For instance, one study employed an 8-week high-intensity cycling program (three sessions per week) and administered 0.4 g·kg^−1^ of SB 30–90 min before each session [139]. Compared to placebo, greater improvements in the lactate threshold and cycling time to fatigue were found for those taking SB, even though total work was matched between groups [139]. Owing to the work-matched design, the benefits are unlikely to stem from acute exercise performance improvements. Instead, these benefits may reflect reduced metabolic disturbance and enhanced mitochondrial respiration in the SB group [139,140]. Another study found greater improvements in Wingate peak power in the group taking 0.2 g·kg^−1^ BM of SB 60–90 min before every exercise session over a 6-week high-intensity interval training program (three sessions per week) [141]. As total work was not matched, the benefits may be due to the accumulation of acute performance enhancements across sessions in the group taking SB.

Although the aforementioned research evidence suggests improved training adaptations with pre-exercise SB, it is not always observed. For example, Siegler et al. [142] did not find additional benefits of SB ingestion across a 10-week period on mechanisms related to explosive power, with a lack of benefit potentially due to the small sample size (*n* = 8). Driller et al. [143] also did not find an additive effect of SB ingestion compared to placebo on 2000 m rowing performance following 4 weeks of high-intensity interval training (2 sessions per week). The lack of an effect may be due to the short duration of 4 weeks. These results may indicate that the effects may depend on factors such as training type or individual responsiveness [142,143] as well as exercise and ingestion protocols.

Summary: Ingestion of SB on the day of competition may improve performance without the need for prior use in training. Still, testing SB supplementation in advance, such as during training, is advisable to minimize side effects, select the protocol or delivery method that works best for the athlete, and increase the likelihood of performance benefits. Preliminary evidence suggests that repeated use of SB during training may enhance adaptations through altered metabolic responses to exercise or accumulated acute performance improvements, although further research is needed.

11. Which sports mainly benefit from sodium bicarbonate supplementation?

An inherent part of sports is often the development of exercise-induced fatigue, which leads to a subsequent decline in performance, an increase in RPE, and an inability to perform the sport at the desired intensity [144]. As such, it would be beneficial to reduce both the development and perception of such fatigue during games, matches, or races. The supplementation with SB, therefore, has been utilized across a range of sports and tasks to reduce this decline and subsequently enhance performance [145]. This extracellular buffer's ergogenic mechanisms are discussed in detail under question 1; however, in essence, it is thought to improve performance via an increase in extracellular pH and a subsequent improved buffering capacity [9,145]. Although SB's primary ergogenic effects are attributed to attenuating the reduction in the intracellular pH during high-intensity exercise, some research also suggests that SB-induced alkalosis may contribute to a reduction in RPE [39,40], which may be particularly important for prolonging exercise performance. While SB has been studied under a variety of interventions, this section focuses on investigations that simulate sport performance, such as through a sport-specific performance test or a TT.

Much of the literature surrounding sports performance and SB supplementation has focused on non-team sports, such as running, cycling, swimming, and rowing. The current body of literature suggests that SB (utilizing a 0.3 g·kg^−1^ BM dose) may aid performance in such sports during high-intensity intermittent protocols, such as 6 × 10 s maximal cycling bouts [54], 6 × 20 m sprints [146], and both 4 × 50 m and 8 × 25 m front crawl swim intervals [82,147]. Moreover, such sports have also been investigated utilizing TT or distance-based protocols, which provide an ecologically valid assessment that reflects the effort and pacing strategy required during a race [96]. In such assessments, the latter half of 2 km rowing performance has been shown to improve after supplementation with a 0.3 g·kg^−1^ BM dose [69], perhaps reflecting an increased ability to maintain a high intensity at the end of an effort with SB. Additionally, performance has been shown to improve over a range of distances following the ingestion SB. Indeed, 4, 16.1, and 40 km cycling TT performance have been shown to benefit from supplementation [32,62,70,114], as well as 800 m, 1500 m, and 3.5 km running TT performance [46,95,148]. All the aforementioned protocols used an 0.3 g·kg^−1^ BM dose; however, the 4 km protocols utilized both an 0.2 and 0.3 g·kg^−1^ BM dose [62,114]. In contrast, there were no performance benefits of an acute 0.3 g·kg^−1^ BM dose of SB during exercise tasks involving: 40 km cycling (when supplemented 60 min pre-exercise, potentially missing the ergogenic window of SB) [92], 2000 m rowing [116,149], and 200 m or 400 m swimming [150]. Such investigations may not have found a benefit of SB ingestion due to the performance level of the participants [149] or the musculature used during the study [150].

During team-based sports that possess a greater skill component than individually competed events, an increase in fatigue may harm decision-making and skill execution [9]. Consequently, SB supplementation may aid performance during such sports, as enhanced extracellular buffering capacity may delay fatigue and preserve motor control, reaction time, and cognitive function [2,122]. However, the effect of SB supplementation on performance in such sports has shown equivocal results. For example, there were no ergogenic effect of an 0.3 g·kg^−1^ BM dose of SB on soccer [105], rugby [73], or water polo performance [151]. However, after ingesting SB (0.3 g·kg^−1^ BM), improvements were found in boxing punch efficacy [65], the number of judo throws [152], repeated-sprint performance in soccer [31] as well as sustained tennis performance [153]. Given these contrasting findings and considering the development of SB delivery methods that reduce GIS, future research should expand to include a variety of team sports and testing protocols to best elucidate how potential benefits and mechanisms differ across contexts.

Overall, the benefits of SB are equivocal across the range of sports; however, ergogenic effects have been observed during activities that are susceptible to high-intensity bouts, whether during a simulated soccer protocol, a 1500 m run, or a 40 km cycling TT [31,32,46,103]. The absence of an ergogenic effect of SB supplementation in certain sports is likely attributable to sport-specific physiological demands. As highlighted herein, SB appears most effective for exercise modalities characterized by high-intensity efforts that elicit substantial metabolic perturbations. In contrast, when a sport does not fundamentally depend on such physiological stressors, SB supplementation is less likely to confer a performance benefit. While much of the literature suggests ergogenic effects during bouts lasting between 30 s and 12 min [2], future research should continue to investigate its benefits across a range of sports and exercise durations. Additionally, the ingestion form and ingestion strategy (further discussed in question #3) must be taken into account when considering supplementation, and athletes should test out various strategies in practice before utilizing the supplement in a competition.

Summary: Supplementing with SB can reduce exercise-induced fatigue and enhance performance, particularly in high-intensity or sustained efforts such as cycling, running, swimming, and rowing, though the results vary across studies and sports. Evidence in skill-based or team-sport simulations is mixed, with some protocols showing improved repeated-sprint ability or skill maintenance and others reporting no significant differences. Overall, the benefits appear most consistent in activities involving intense bouts lasting 30 s to 12 min, but responses depend on the sport, protocol, and ingestion strategy.

12. Should one worry about the sodium dose?

The ingestion of SB carries a risk of a high daily sodium load that athletes and practitioners should consider as part of the ingestion strategy. Specifically, for a 70 kg athlete, a 0.3 g·kg^−1^ BM SB dose would equate to 21 g of total SB, where ~5.75 g is sodium. For context, this equates to more than double the recommended daily sodium intake of 2.4 g by the UK National Health Service for the general population [154]. Such high amounts of sodium may place the athlete's health at risk through increased renal workload and exacerbate GIS which could negatively influence exercise performance [4,155,156]. Indeed, in one study administering a single 0.3 g·kg^−1^ BM SB dose a significant increase in diastolic blood pressure was reported during rest, exercise, and recovery in healthy adults (~3–4 mmHg); however, this was not interpreted as being a clinically relevant change [4]. Based on this study, it is intuitive to suggest that chronically this might cause issues with physiological function. It is worth noting, however, that these studies are scant and have only been conducted in healthy individuals, not in athletes with high training loads. This is problematic, as athletes typically have a higher sodium turnover due to increased sodium sweat losses and therefore the high sodium load may not present an issue. On the balance of the limited evidence, a delicate interplay between the sodium intake (via diet and SB ingestion) and sodium losses (via exercise) needs to be considered to ensure that ergogenic benefits can be obtained without any potential negative physiological outcomes.

Although concerns about SB often focus on its sodium load, its physiological impact can also be understood through its effects on strong-ion balance [23]. Albeit discussed more in question #1, SB ingestion increases extracellular Na⁺ and reduces Cl^−^, shifting the SID and contributing to the alkalosis that supports H⁺ efflux [24]. These ion shifts also interact with K⁺ regulation, which is central to maintaining membrane excitability [26,28]. This extracellular buffer has been shown to reduce the rise in extracellular K⁺ during high-intensity exercise by likely preserving intracellular K⁺ [29,32]; however, this is not always observed [104,157]. Still, rather than posing a risk due to sodium alone, SB influences a broader strong-ion response that helps preserve muscle excitability and may underpin its ergogenic effects.

Practically, the sodium load should be considered as part of planning SB ingestion strategies. The priority actions alongside SB ingestion would be to monitor blood pressure and body mass changes (especially for weight category sports), GIS, and nutrition intake monitoring for sodium and potassium intake (i.e. via dietary recall). Of course, the performance responses should also be monitored to ensure that the SB is creating an ergogenic response. As most of these measurements are taken in practice settings with high performance athletes, it should not create too much of a measurement burden for both the practitioner and/or athlete. Since SB can also be used as a hyperhydration strategy and increase plasma volume [14,158], it is also important to monitor athletes' use of sodium-based electrolyte products, as these will add further total sodium and are unlikely to be required given the already high sodium intake when using SB. These procedures will be more important for athletes who have a low sodium turnover, which is often seen in weightlifting type sports, whereas for endurance athletes, there is less of a risk of negative consequences from SB use. These procedures may be important during a race weekend, where there are heats and finals, and therefore, repeated dosing remains attractive to athletes. We would advise a cautious approach knowing that SB can improve multiple bouts of exercise with a recovery window of between 15 and 90 min [126].

Summary: ingesting SB risks exceeding the recommended daily sodium load and consequently increases the likelihood of raising GIS and renal function pressure. At present, there is no data that suggests increased blood pressure in athletes following SB use. However, given that some athletes have a high sodium turnover (i.e. endurance athletes) due to heightened exercise demands, it is possible that the elevated sodium load associated with a typical dose of SB (0.3 g·kg^−1^) may not pose a significant physiological issue. Nevertheless, greater attention to total sodium intake is warranted for athletes with lower sodium turnover (i.e. weightlifters), and repeated SB dosing should be done cautiously. Overall, where possible, sport practitioners should aim to monitor nutrition intake, GIS, blood pressure, and body mass as a means of managing the potential sodium-related risks associated with SB. More research is needed on the chronic use of SB in athletes for training and repeated competition events/days.

13. How does extracellular buffering compare to intracellular buffering agents such as beta-alanine?

Intra- and extracellular buffering systems protect against excessive accumulation of H^+^ and subsequent decreases in muscle pH during high-intensity exercise [159]. Beta-alanine (BA) is a non-essential amino acid that acts as the rate-limiting precursor to carnosine synthesis [160,161]. Carnosine contains an imidazole ring (pKa: 6.83, close to the physiological pH of muscle), which allows it to neutralize H^+^ in muscles during intense exercise [162]. Chronic BA supplementation increases the intramuscular carnosine concentration, typically 18–26 mmol·kg^−1^ dry weight in human skeletal muscle [163], and acts as an intracellular buffer [164]. Conversely, SB serves as an extracellular buffering agent, as its dissociation increases the blood bicarbonate concentration, which permits greater efflux of H^+^ out of the muscle cell that maintains intramuscular pH [17]. Despite the similarities in the roles of BA and SB as buffers, their optimal dosing strategies differ. Chronic BA supplementation of 4–6 g·day^−1^ for ≥4 weeks is recommended [165]. Meanwhile, SB can be taken acutely (0.2–0.3 g·kg^−1^ BM), with the pre-exercise ingestion time varying depending on the form of administration (discussed in questions 3 and 7). From a practical perspective, SB can be strategically scheduled on an event day, whereas BA requires early, long-term planning consisting of daily supplementation for weeks, with no additional considerations on the day of competition.

While carnosine content typically increases following BA supplementation, inter-individual variability likely exists. Baguet et al. [164] observed high- and low-responders after 4.8 g·day^−1^ BA for 5–6 weeks, with carnosine washout periods ranging between 6 and 15 weeks. Inter-individuality of responses for BA might be related to muscle fiber composition or athletic status [166]. Meanwhile, the efficacy of SB may range due to the large degree of variability in bicarbonate absorption rates [55,167], genetic variations affecting MCT transporters and subsequent H^+^ removal [12,168], and GIS [43], thereby affecting subsequent performance.

Indeed, BA and SB have established side-effects that differ. For example, BA is commonly known to cause paresthesia or tingling [165]. Symptoms typically appear when BA is given in a non-sustained release form [160] and disappear 60–90 min after supplementation [169]. Currently, there is no evidence to suggest that side-effects are harmful at the aforementioned doses administered [165,170,171]. Alternatively, SB ingestion, especially when administered in a non-gastro-resistant form (simple capsule form or solution), may cause GIS, which can sometimes persist for ~90–120 min post-ingestion [73]. However, gastro-resistant forms of SB may reduce GIS substantially [7,59].

The duration of exercise that benefits most from BA or SB differs slightly. While both supplements have ergogenic effects on high-intensity exercise lasting 30 s to 10 min [2,138], SB may be more effective for endurance activities, in part owing to mechanisms outside buffering, such as modulating hydration and strong ion changes [14,172]). Additionally, meta-analytical data suggests that BA appears to produce more consistent benefits for tests of exercise capacity than exercise performance [138,173], with modest improvements typically observed for exercise lasting up to 25 min [165], but research investigating longer durations is limited [165,174]. Regarding SB supplementation, high-intensity exercise < 30 s may not induce sufficient H⁺ accumulation to justify SB supplementation, and the rapid intramuscular generation of H⁺ may surpass the transport capacity of MCTs, thereby limiting efflux into the extracellular space (discussed in more detail in question 5) [3,168]. Similarly, longer exercise duration examinations are lacking, though some investigations have observed improvements during prolonged running [89,95] and cycling [32,70] or across simulated team sport match scenarios [31,68].

Despite BA and SB acting on different buffering systems, implying that greater effects might be observed by combining supplementation [138], evidence is equivocal whether co-ingestion of chronic BA and acute SB produces an additive benefit. Several studies have examined the combined effects of BA and SB, with mixed outcomes. Bellinger et al. [175] reported only a minimal additive effect on 4-min cycling (3.3% vs. 3.1% with SB alone), while Mero et al. [81] did not find a benefit beyond SB in sprint swimming. Similarly, Sale et al. [176] showed that BA, SB, and their combination all increased cycling TTE, but without significant differences between them; despite this, their combination resulted in a 16.2% change, whereas SB and BA had 6.5% and 12.1% changes, respectively. In contrast, Tobias et al. [177] observed an additive effect during repeated upper-body Wingate tests, with BA + SB improving total work by 14%, compared to 7% and 8% changes observed with BA and SB alone. More recently, a systematic review and meta-analysis [178] reported that there was no ergogenic effect for BA or SB individually but did find a benefit when the two were combined. Taken together, while some protocols and pooled evidence suggest a potential additive benefit, the overall body of research remains inconsistent. Discrepancies in such results may originate from differences in protocol design and outcome measures, as well as small sample sizes and the small scale of work in the area.

Both BA and SB enhance the intracellular and extracellular buffering capacity during intense exercise, though their dosing strategies and side effects differ. Although BA requires chronic loading and may cause paresthesia, it offers a longer-term option for athletes who are susceptible to GIS. Conversely, SB can be administered as an acute dose on a race/competition day but must first be trialed on an individual basis. Ultimately, both buffering agents have been shown to be effective ergogenic aids. Therefore, the choice of buffering aid, or their combination, should be guided by the athlete's competition demands, tolerance, and event duration, with empirical evidence supporting its use during high-intensity exercise lasting 30 s–10 min [2,138], with a potential increased scope for the utilization of SB during endurance activities.

Summary: Beta-alanine and SB both enhance buffering during high-intensity exercise, but BA works intracellularly through chronic loading of muscle carnosine, whereas SB acutely increases extracellular bicarbonate. Each supplement has distinct side effects and shows performance benefits, mainly for high-intensity efforts lasting 30 s–10 min, with SB potentially extending to longer endurance events. Combining them may offer additive effects, but more work is needed in the area to fully elucidate their effects.

14. Is there a possible placebo effect with sodium bicarbonate?

The established mechanisms of SB are discussed in greater detail in question #1; however, outside of its role as an extracellular buffer, increasing research evidence suggests that at least part of SB's ergogenic effects may be attributable to psychological expectancy or athletes' beliefs about whether this alkalizing agent will help or hinder performance [2,179–181]. This placebo component has been demonstrated across several study designs, ranging from classical balanced placebo paradigms to modern mediation analyses.

Early work by McClung & Collins [180] highlighted the substantial influence of expectancy on exercise performance, using a balanced placebo design in trained runners. These authors demonstrated that participants who believed that they had ingested SB improved their 1000 m TT-run performance to a degree comparable to when they *actually* consumed the supplement. Conversely, when SB was administered but participants were led to believe that they had not received the extracellular buffer, no performance enhancement was observed. These findings suggest that expectancy alone can produce an ergogenic response similar in magnitude to the physiological effects of SB, whereas the absence of positive expectancy largely eliminates the benefit. This interpretation aligns with a recent meta-analysis by Marticorena et al. [182], who reported a small effect size but statistically significant performance improvement following placebo ingestion (ES_0.5_ = 0.09; 95% credible interval [(CrI)], 0.01–0.17) and estimated that approximately 25% (75% CrI, 16% to 35%) of the performance gains typically attributed to buffering agents, including SB, may arise from placebo-related mechanisms rather than physiological buffering.

More recent research has employed designs that enable clearer separation of the psychological and physiological components of SB's effects on performance. Gurton et al. [179] examined both expectancy and changes in blood bicarbonate during TTE cycling. While SB improved performance in both solution and capsule forms, participants exhibited higher expectations of benefit when it was administered in solution (median: 3.5 AU vs. 2.5 AU, Z = 2.14, *p* = 0.033), likely due to the “poor” taste of solution beverages [71]. This heightened expectancy predicted greater effort, which in turn produced a larger decline in blood bicarbonate during exercise (reflecting greater extracellular buffering use) (2.7 ± 2.1 mmol·L^−1^, *p* = 0.001). Mediation analyses revealed that expectancy partially explained performance improvement, suggesting that psychological and physiological mechanisms interact rather than operate independently.

The influence of belief becomes even clearer in research that manipulates expectancy directly. In a placebo–nocebo design, Zagatto et al. [181] informed participants that a supplement would have either ergogenic, inert, or harmful effects. Under all conditions, SB induced metabolic alkalosis, but performance improved only when athletes were told that the supplement would help, even when performance changes occurred under placebo ingestion. Nocebo instructions eliminated performance benefits despite inducing alkalosis to a degree suggested to improve exercise performance, demonstrating that expectancy can override physiological readiness in short-duration, high-intensity exercise. Similar findings have been reported with SHAM placebos. Higgins & Shabir [183] showed that a SHAM placebo designed to mimic SB-related GIS (SHAM: 0.1 g·kg^−1^ NaCl, 4 mL·kg^−1^ carbonated water, and 1 mL·kg^−1^ sugar-free squash) improved cycling TTE by 9.5% when compared to a placebo (PLA: 0.1 g·kg^−1^ NaCl, 4 mL·kg^−1^ tap water, and 1 mL·kg^−1^ sugar-free squash). Collectively, these studies indicate that psychological expectancy can meaningfully influence performance and, at times, potentially override the physiological ergogenic mechanisms of SB ingestion in short, high-intensity exercise.

Taken together, these findings support the position outlined in the ISSN Position Stand [2], which states that while SB reliably increases blood bicarbonate and enhances buffering capacity, “a portion of the ergogenic effect seems to be placebo-driven”. Expectancy, belief, GIS, taste cues, and blinding effectiveness all contribute to variability across the available studies and real-world outcomes. This does not diminish the physiological validity of SB supplementation but instead highlights the complex interplay between the mind and body in externally valid performance settings. For practitioners, these findings emphasize the importance of appropriate education, individualized supplementation strategies, and avoiding unintentional nocebo effects when discussing potential side effects.

Summary: The ingestion of SB enhances performance, but part of this effect appears to stem from psychological expectancy rather than physiological buffering alone. Studies using balanced-placebo, mediation, and expectancy-manipulation designs show that simply believing one has taken SB can produce performance improvements comparable to those of the supplement itself, while negative expectations can eliminate benefits even when alkalosis is achieved. This evidence suggests that placebo-related mechanisms meaningfully contribute to the variability in SB's ergogenic effects. The form of SB also likely influences participant blinding and placebo effects, where SB in capsules reduces placebo responses when compared to aqueous solution due to increased uncertainty. Researchers and practitioners should remain cautious of the potential placebo-related effects of SB given that the ingestion form can influence participant expectations, but more research is needed on these effects with GIS limiting SB ingestion forms.

15. Are the ergogenic effects of sodium bicarbonate sex-specific?

While the potential effects of SB for high-intensity efforts are well understood [2], one question that arises is whether this extracellular buffer's ergogenic benefits are sex-specific. The majority of research investigating SB ingestion on exercise performance has been largely conducted in biological male samples. The most recent and comprehensive position stand on this subject highlighted that from 124 studies reviewed, only eight focused on female participants, with a further 14 studies involving mixed cohorts [2]. This contrasts with 101 studies focusing on solely male participants. In context, a total of 170 female participants were included in the studies reviewed involving SB, compared to 1246 male participants. It is therefore evident that broader research into the effects of SB for women is clearly required, as supported elsewhere [61].

Doses of 0.2–0.5 g·kg^−1^ BM are well established to enhance short-term high-intensity exercise, including peak anaerobic power and anaerobic capacity [2,184], as well as muscular endurance in activities lasting up to 12 min [2] through H^+^ buffering. Although the ergogenic benefits of SB ingestion have been demonstrated in women, it is reasonable to suggest that women may respond differently to extracellular buffering given established physiological sex-based differences. Specifically, women typically exhibit lower overall muscle mass [185], a lower proportion of type II muscle fibers [186,187], and reduced glycolytic capacity associated with diminished glycolytic enzyme activity [188] which likely contributes to decreased high-intensity exercise performance when compared to men [189]. Given these differences, it is plausible to suggest that women may have a reduced ergogenic response to SB ingestion in comparison to men. Furthermore, in a recent study, significant sex-based differences in blood bicarbonate concentrations were observed between men and women, even following the ingestion of SB at a relatively small dose of 0.15 g·kg fat free mass (FFM^−1^) [190]. For females, doses of 0.25 g·kg FFM^−1^ were required to reach an increase in blood bicarbonate, which is considered potentially ergogenic (5 mmol·L^−1^), whereas male participants reached this same potentially ergogenic threshold despite using a smaller dose [190]. This also corresponded with improved mean and peak sprint power for male participants, potentially highlighting that ergogenic buffering via SB may lead to a greater performance-enhancing effect in men. A similar finding has also been reported for high intensity performance in wrestlers [182], again indicating that low dose SB may be less effective in female athletes.

Furthermore, it is possible sex-specific GIS differences may influence the absorption and metabolism of SB which may suggest a reduced buffering capacity. Recently, it has been reported that the GIS (observed with increasing dose) may be greater in women, which may impact both nutrient absorption and relative tolerance to SB [202]. This may be particularly relevant when considering that a SB dose of 0.3 g·kg^−1^ is recommended for acute ergogenic benefits [138], and higher total daily intakes (0.4–0.5 g·kg^−1^) may be pertinent when considering multi-day protocols. However, further research is warranted to corroborate such mechanisms.

It is important to highlight that despite the potential for sex-related differences, ergogenic effects of SB have still been observed in women with doses ranging from 0.2 to 0.4 g·kg^−1^ BM [68,74,191,192]. For example, following maximal effort (60 s cycling), consumption of 0.3 g·kg^−1^ SB improved both total work (26.9 ± 0.6 vs. 24.5 ± 0.7 kJ) and peak power (769.4 ± 28 vs. 727.1 ± 33 W) compared to placebo in moderately trained women [191]. Similarly, through the use of enteric-coated SB, a dose of 0.3 g·kg^−1^ BM resulted in a ~2% improvement in 2 km rowing performance (~15.6 s faster) in CrossFit® female athletes [74]. Similar ergogenic benefits have been shown in extended short (3-day) and longer-term (8-week) supplementation periods [139,192] impacting high intensity efforts (sprint times, jump height, circuit times), lactate threshold and time to fatigue. However, such findings have not been replicated elsewhere in competitive female athletes [110,150], although the latter study may be confounded by the inclusion of a mixed-sex cohort.

Summary: Most of the research with female, as well as male participants, would suggest that SB is ergogenic for high-intensity exercise performance in both sexes. However, additional research is needed to further explore the effect of SB ingestion on biological female athletes. Future research should explore the effects of SB ingestion on metabolism and exercise performance across menstrual phases to explore whether it affects extracellular buffering capacity, and consequently, the ergogenic effects of SB.

## 2. Conclusion

Based on our evidence-based evaluation of the literature, we conclude that:

- Sodium bicarbonate enhances high-intensity exercise performance primarily by increasing the extracellular buffering capacity, which subsequently promotes greater H⁺ efflux, thereby preserving the intramuscular pH and key metabolic and contractile processes.
- There is evidence that SB supplementation can result in adverse effects, primarily involving the GIS. These effects are more evident at a dose of ≥0.3 g·kg^−1^ BM as well as in non-gastro-resistant formulations (i.e. aqueous solution, gelatin/vegetarian capsules).
- Regardless of the ingestion form, SB ingestion increases blood bicarbonate and improves high-intensity exercise performance; however, side effects are exacerbated when SB interacts with stomach acid, particularly in non-gastro-resistant forms (e.g. solution, gelatin capsules). Therefore, selecting a form of SB that reduces the side effects associated with this extracellular buffer will enhance practicality and will likely optimize performance.
- Most evidence suggests that SB ingestion enhances high-intensity exercise performance up to 10 min in duration, with reduced benefits during very short bouts of exercise (<30 s) likely owing to an attenuation in proton buffering.
- Sodium bicarbonate has predominantly been examined in short-duration high-intensity exercise, yet an increasing body of evidence suggests that it may also improve prolonged high-intensity performance.
- There are plausible uses for SB ingestion in extreme environments, namely, altitude and heat. It is likely that SB ingestion at altitude will provide supplemental bicarbonate to alleviate the potential reduction in bicarbonate commonly observed at altitude; however, its ergogenic effects in this environment are less clear.
- The preponderance of evidence suggests that consuming SB 60–120 min pre-exercise is an effective strategy to observe an ergogenic effect, but this can be specific to the ingestion form.
- Post‑exercise SB supplementation may enhance recovery by accelerating blood acid‑base restoration, reducing cellular stress, and improving subsequent performance, particularly when sufficient recovery time is available between exercise (e.g. between heats and finals).
- Sodium bicarbonate supplementation on the day of competition may improve performance without the need for prior use in training. However, experimentation during training is recommended to identify optimal supplementation protocols. Preliminary evidence suggests that repeated use of SB during training may enhance adaptations through altered metabolic responses to exercise or accumulated acute performance improvements, although further research is needed.
- Sodium bicarbonate supplementation can reduce exercise-induced fatigue and enhance performance, particularly in high-intensity or sustained efforts such as cycling, running, swimming, and rowing, though results vary across studies and sports.
- High doses of SB, such as 0.3 g·kg^−1^ BM, can deliver high levels of sodium (i.e. about 5.75 g for a 70 kg athlete), which likely causes athletes to exceed the recommended daily intake. This may cause some renal function strain, and ingestion form-related GIS. At present, the effects on blood pressure are not adequately evidenced, so caution is warranted; however, in otherwise healthy endurance athletes, this is unlikely to be an issue.
- Beta-alanine and SB both enhance buffering during high-intensity exercise, but BA works intracellularly through chronic loading of muscle carnosine, whereas SB acutely increases extracellular bicarbonate. Each has distinct side effects and shows performance benefits mainly for efforts lasting 30 s–10 min, with SB potentially extending to longer endurance events.
- There is evidence that suggests that placebo-related mechanisms meaningfully contribute to the variability in SB's ergogenic effects.
- SB improves exercise performance in males and females. However, given that the majority of currently available research is conducted among male samples, future research is needed to explore different aspects of SB supplementation among females.

## References

[1] Dennig H , Talbott JH , Edwards HT , et al. Effect of acidosis and alkalosis upon capacity for work. J Clin Invest. 1931;9(4):601–613. doi: 10.1172/JCI100324 16693953 PMC435718

[2] Grgic J , Pedisic Z , Saunders B , et al. International society of sports nutrition position stand: sodium bicarbonate and exercise performance. J Int Soc Sports Nutr. 2021;18(1):61. doi: 10.1186/s12970-021-00458-w 34503527 PMC8427947

[3] de Oliveira LF , Dolan E , Swinton PA , et al. Extracellular buffering supplements to improve exercise capacity and performance: a comprehensive systematic review and meta-analysis. Sports Med. 2022;52(3):505–526. doi: 10.1007/s40279-021-01575-x 34687438

[4] Kahle LE , Kelly PV , Eliot KA , et al. Acute sodium bicarbonate loading has negligible effects on resting and exercise blood pressure but causes gastrointestinal distress. Nutr Res. 2013;33(6):479–486. doi: 10.1016/j.nutres.2013.04.009 23746564 PMC3680785

[5] Hilton NP , Leach NK , Craig MM , et al. Enteric-coated sodium bicarbonate attenuates gastrointestinal side-effects. Int J Sport Nutr Exerc Metab. 2020 cited 2025 Dec 16]; doi: 10.1123/ijsnem.2019-0151 31751936

[6] Dalle S , Koppo K , Hespel P . Sodium bicarbonate improves sprint performance in endurance cycling. J Sci Med Sport. 2021;24(3):301–306. doi: 10.1016/j.jsams.2020.09.011 34756350

[7] Gough LA , Sparks SA . The effects of a carbohydrate hydrogel system for the delivery of bicarbonate mini-tablets on Acid–Base buffering and gastrointestinal symptoms in resting well-trained Male cyclists. Sports Med Open. 2024;10:17. doi: 10.1186/s40798-024-00684-x 38356036 PMC10866843

[8] Carr AJ , Hopkins WG , Gore CJ . Effects of acute alkalosis and acidosis on performance: a meta-analysis. Sports Med. 2011;41(10):801–814. doi: 10.2165/11591440-000000000-00000 21923200

[9] McNaughton LR , Siegler J , Midgley A . Ergogenic effects of sodium bicarbonate. Curr Sports Med Rep. 2008;7(4):230–236. doi: 10.1249/JSR.0b013e31817ef530 18607226

[10] Bishop D , Edge J , Davis C , et al. Induced metabolic alkalosis affects muscle metabolism and repeated-sprint ability. Med Sci Sports Exerc. 2004;36(5):807–813. doi: 10.1249/01.MSS.0000126392.20025.17 15126714

[11] Hollidge-Horvat MG , Parolin ML , Wong D , et al. Effect of induced metabolic alkalosis on human skeletal muscle metabolism during exercise. Am J Physiol Endocrinol Metab. 2000;278(2):E316–329. doi: 10.1152/ajpendo.2000.278.2.E316 10662717

[12] Heibel AB , Perim PHL , Oliveira LF , et al. Time to optimize supplementation: modifying factors influencing the individual responses to extracellular buffering agents. Front Nutr [Internet]. 2018;5:35. doi: 10.3389/fnut.2018.00035. cited 2025 Sept 22]. 29868599 PMC5951986

[13] Spriet LL , Gledhill N , Froese AB , et al. Effect of graded erythrocythemia on cardiovascular and metabolic responses to exercise. J Appl Physiol (1985). 1986;61(5):1942–1948. doi: 10.1152/jappl.1986.61.5.1942 3781999

[14] Siegler JC , Carr AJ , Jardine WT , et al. The hyperhydration potential of sodium bicarbonate and sodium citrate. Int J Sport Nutr Exercise Metab. 2022;32:74–81. doi: 10.1123/ijsnem.2021-0179 34875625

[15] Westerblad H . Acidosis is not a significant cause of skeletal muscle fatigue. Med Sci Sports Exerc. 2016;48(11):2339–2342. doi: 10.1249/MSS.0000000000001044 27755383

[16] Westerblad H , Allen DG . Changes of intracellular pH due to repetitive stimulation of single fibres from mouse skeletal muscle. J Physiol. 1992;449:49–71. doi: 10.1113/jphysiol.1992.sp019074 1522520 PMC1176067

[17] Requena B , Zabala M , Padial P , et al. Sodium bicarbonate and sodium citrate: ergogenic aids? J Strength Cond Res. 2005;19(1):213–224. doi: 10.1519/00124278-200502000-00036 15705037

[18] Fitts RH . The cross-bridge cycle and skeletal muscle fatigue. J Appl Physiol (1985). 2008;104(2):551–558. doi: 10.1152/japplphysiol.01200.2007 18162480

[19] Fitts RH . The role of acidosis in fatigue: pro perspective. Med Sci Sports Exerc. 2016;48(11):2335–2338. doi: 10.1249/MSS.0000000000001043 27755382

[20] Donaldson SK , Hermansen L , Bolles L . Differential, direct effects of H^+^ on Ca2^+^ -activated force of skinned fibers from the soleus, cardiac and adductor magnus muscles of rabbits. Pflugers Arch. 1978;376(1):55–65. doi: 10.1007/BF00585248 30066

[21] Debold EP , Beck SE , Warshaw DM . Effect of low pH on single skeletal muscle myosin mechanics and kinetics. Am J Physiol Cell Physiol. 2008;295(1):C173–179. doi: 10.1152/ajpcell.00172.2008 18480297 PMC2493560

[22] Messonnier L , Kristensen M , Juel C , et al. Importance of pH regulation and lactate/H^+^ transport capacity for work production during supramaximal exercise in humans. J Appl Physiol (1985). 2007;102(5):1936–1944. doi: 10.1152/japplphysiol.00691.2006 17289910

[23] Stewart PA . Modern quantitative acid–base chemistry. Can J Physiol Pharmacol. 1983;61(12):1444–1461. doi: 10.1139/y83-207 6423247

[24] Heigenhauser GJ , Lindinger MI . The total ionic status of muscle during intense exercise. Adv Exp Med Biol. Vol. 227 1988. p. 237–242. doi: 10.1007/978-1-4684-5481-9_21 3381699

[25] Lühker O , Berger MM , Pohlmann A , et al. Changes in acid-base and ion balance during exercise in normoxia and normobaric hypoxia. Eur J Appl Physiol. 2017;117(11):2251–2261. doi: 10.1007/s00421-017-3712-z 28914359 PMC5640730

[26] Cairns SP , Lindinger MI . Do multiple ionic interactions contribute to skeletal muscle fatigue? J Physiol. 2008;586(17):4039–4054. doi: 10.1113/jphysiol.2008.155424 18591187 PMC2652190

[27] Clausen T . Na^+-^k^+^ pump regulation and skeletal muscle contractility. Physiol Rev. 2003;83(4):1269–1324. doi: 10.1152/physrev.00011.2003 14506306

[28] Sejersted OM , Sjøgaard G . Dynamics and consequences of potassium shifts in skeletal muscle and heart during exercise. Physiol Rev. 2000;80(4):1411–1481. doi: 10.1152/physrev.2000.80.4.1411 11015618

[29] Gough LA , Deb SK , Brown D , et al. The effects of sodium bicarbonate ingestion on cycling performance and acid base balance recovery in acute normobaric hypoxia. J Sports Sci. 2019;37(13):1464–1471. doi: 10.1080/02640414.2019.1568173 30668281

[30] Gough LA , Rimmer S , Sparks SA , et al. Post-exercise supplementation of sodium bicarbonate improves acid base balance recovery and subsequent high-intensity boxing specific performance. Front Nutr. 2019;6:155. doi: 10.3389/fnut.2019.00155 31632978 PMC6779834

[31] Gurton WH , Gough LA , & Siegler JC , et al. Oral but Not Topical Sodium Bicarbonate Improves Repeated Sprint Performance During Simulated Soccer Match Play Exercise in Collegiate Athletes. [cited 2025 Aug 19]. 2024. doi: 10.1123/ijsnem.2024-0059 39222921

[32] Shannon ES , Regnier A , Dobson B , et al. The effect of sodium bicarbonate mini-tablets ingested in a carbohydrate hydrogel system on 40 km cycling time trial performance and metabolism in trained Male cyclists. Eur J Appl Physiol. 2024;124(12):3671–3682. doi: 10.1007/s00421-024-05567-3 39068627 PMC11568979

[33] Newbury JW , Cole M , Kelly AL , et al. Neither an individualised nor a standardised sodium bicarbonate strategy improved performance in high-intensity repeated swimming, or a subsequent 200 m swimming time trial in highly trained female swimmers. Nutrients. 2024;16(18):3123. doi: 10.3390/nu16183123 39339723 PMC11434820

[34] Sostaric SM , Skinner SL , Brown MJ , et al. Alkalosis increases muscle K^+^ release, but lowers plasma [K^+^] and delays fatigue during dynamic forearm exercise. J Physiol. 2006;570(Pt 1):185–205. doi: 10.1113/jphysiol.2005.094615 16239279 PMC1464289

[35] Siegler JC , Midgley AW , Polman RCJ , et al. Effects of various sodium bicarbonate loading protocols on the time-dependent extracellular buffering profile. J Strength Cond Res. 2010;24(9):2551–2557. doi: 10.1519/JSC.0b013e3181aeb154 20040895

[36] Fraser SF , Li JL , Carey MF , et al. Fatigue depresses maximal in vitro skeletal muscle na^+-^k^+-^atpase activity in untrained and trained individuals. J Appl Physiol. 2002;93(5):1650–1659. doi: 10.1152/japplphysiol.01247.2001 12381750

[37] Robertson RJ , Falkel JE , Drash AL , et al. Effect of induced alkalosis on physical work capacity during arm and leg exercise. Ergonomics. 1987;30(1):19–31. doi: 10.1080/00140138708969674 3030725

[38] Swank AM , Robertson RJ . Effect of induced alkalosis on perception of exertion during exercise recovery. J Strength Condit Res. 2002;16(4):491. 12423176

[39] Swank A , Robertson RJ . Effect of induced alkalosis on perception of exertion during intermittent exercise. J Appl Physiol (1985). 1989;67(5):1862–1867. doi: 10.1152/jappl.1989.67.5.1862 2557322

[40] Siegler JC , Marshall P . The effect of metabolic alkalosis on central and peripheral mechanisms associated with exercise-induced muscle fatigue in humans. Exp Physiol. 2015;100(5):519–530. doi: 10.1113/EP085054 25727892

[41] Siegler JC , Marshall P , Pouslen MK , et al. The effect of pH on fatigue during submaximal isometric contractions of the human calf muscle. Eur J Appl Physiol. 2015;115(3):565–577. doi: 10.1007/s00421-014-3027-2 25351788

[42] Carr AJ , Slater GJ , Gore CJ , et al. Effect of sodium bicarbonate on [HCO_3−_], pH, and gastrointestinal symptoms. Int J Sport Nutr Exerc Metab. 2011;21(3):189–194. doi: 10.1123/ijsnem.21.3.189 21719899

[43] Saunders B , Sale C , Harris RC , et al. Sodium bicarbonate and high-intensity-cycling capacity: variability in responses. Int J Sports Physiol Perform. 2014;9(4):627–632. doi: 10.1123/ijspp.2013-0295 24155093

[44] Gurton WH , Gough LA , Sparks SA , et al. Sodium bicarbonate ingestion improves time-to-exhaustion cycling performance and alters estimated energy system contribution: a dose-response investigation. Front Nutr. 2020;7:154. doi: 10.3389/fnut.2020.00154 33015125 PMC7506131

[45] McNaughton LR . Bicarbonate ingestion: effects of dosage on 60 s cycle ergometry. J Sports Sci. 1992;10(5):415–423. doi: 10.1080/02640419208729940 1331493

[46] Bird SR , Wiles J , Robbins J . The effect of sodium bicarbonate ingestion on 1500‐m racing time. J Sports Sci. 1995;13(5):399–403. doi: 10.1080/02640419508732255 8558626

[47] Froio de Araujo Dias G , da Eira Silva V , de Salles Painelli V , et al. (In)Consistencies in responses to sodium bicarbonate supplementation: a randomised, repeated measures, counterbalanced and double-blind study. PLoS One. 2015;10(11):e0143086. doi: 10.1371/journal.pone.0143086 26574755 PMC4648485

[48] Siegler JC , Marshall PWM , Bray J , et al. Sodium bicarbonate supplementation and ingestion timing: does it matter?. J Strength Cond Res. 2012;26(7):1953–1958. doi: 10.1519/JSC.0b013e3182392960 21964428

[49] Durkalec-Michalski K , Zawieja EE , Podgórski T , et al. The effect of a new sodium bicarbonate loading regimen on anaerobic capacity and wrestling performance. Nutrients. 2018;10(6):697. doi: 10.3390/nu10060697 29848993 PMC6024820

[50] Durkalec-Michalski K , Zawieja EE , Podgórski T , et al. The effect of chronic progressive-dose sodium bicarbonate ingestion on CrossFit-like performance: a double-blind, randomized cross-over trial. PLoS One. 2018;13(5):e0197480. doi: 10.1371/journal.pone.0197480 29771966 PMC5957406

[51] Gough LA , Sparks SA . The effects of a novel sodium bicarbonate ingestion system on repeated 4 km cycling time trial performance in well-trained Male cyclists. Sports Med. 2024;54(12):3199–3210. doi: 10.1007/s40279-024-02083-4 39122982 PMC11608388

[52] Franc A , Vetchý D , Fülöpová N . Commercially available enteric empty hard capsules, production technology and application. Pharmaceuticals (Basel). 2022;15(11):1398. doi: 10.3390/ph15111398 36422528 PMC9696354

[53] Gough LA , Deb SK , Sparks A , et al. The reproducibility of 4-km time trial (TT) performance following individualised sodium bicarbonate supplementation: a randomised controlled trial in trained cyclists. Sports Med - Open. 2017;3(1):34. doi: 10.1186/s40798-017-0101-4 28936625 PMC5608656

[54] Miller P , Robinson AL , Sparks SA , et al. The effects of novel ingestion of sodium bicarbonate on repeated sprint ability. J Strength Cond Res. 2016;30(2):561–568. doi: 10.1519/JSC.0000000000001126 26815179

[55] Farias De Oliveira L , Saunders B , Yamaguchi G , et al. Is individualization of sodium bicarbonate ingestion based on time to peak necessary? Med Sci Sports Exercise. 2020;52(8):1801–1808. doi: 10.1249/MSS.0000000000002313 32102054

[56] Jones RL , Stellingwerff T , Artioli GG , et al. Dose-response of sodium bicarbonate ingestion highlights individuality in time course of blood analyte responses. Int J Sport Nutr Exerc Metab. 2016;26(5):445–453. doi: 10.1123/ijsnem.2015-0286 27098290

[57] Middlebrook I , Peacock J , Tinnion DJ , et al. Capsule size alters the timing of metabolic alkalosis following sodium bicarbonate supplementation. Front Nutr [Internet]. 2021;8: doi: 10.3389/fnut.2021.634465 [cited 2025 Nov 20]. PMC7933015 33681279

[58] Hilton NP , Leach NK , Sparks SA , et al. A novel ingestion strategy for sodium bicarbonate supplementation in a delayed-release form: a randomised crossover study in trained males. Sports Med Open. 2019;5(1):4. doi: 10.1186/s40798-019-0177-0 30680463 PMC6346694

[59] Hilton NP , Leach NK , Hilton MM , et al. Enteric-coated sodium bicarbonate supplementation improves high-intensity cycling performance in trained cyclists. Eur J Appl Physiol. 2020;120(7):1563–1573. doi: 10.1007/s00421-020-04387-5 32388584 PMC7295736

[60] Peart DJ , Siegler JC , Vince RV . Practical recommendations for coaches and athletes: a meta-analysis of sodium bicarbonate use for athletic performance. J Strength Cond Res. 2012;26(7):1975–1983. doi: 10.1519/JSC.0b013e3182576f3d 22505127

[61] Saunrsde B , de Oliveira LF , Dolan E , et al. Sodium bicarbonate supplementation and the female athlete: a brief commentary with small scale systematic review and meta-analysis. Eur J Sport Sci. 2022;22(5):745–754. doi: 10.1080/17461391.2021.1880649 33487131

[62] Gough LA , Deb SK , Sparks SA , et al. Sodium bicarbonate improves 4 km time trial cycling performance when individualised to time to peak blood bicarbonate in trained Male cyclists. J Sports Sci. 2018;36(15):1705–1712. doi: 10.1080/02640414.2017.1410875 29183257

[63] Lavender G , Bird SR . Effect of sodium bicarbonate ingestion upon repeated sprints. Br J Sports Med. 1989;23(1):41–45. doi: 10.1136/bjsm.23.1.41 2730998 PMC1478644

[64] McNaughton LR . Sodium bicarbonate ingestion and its effects on anaerobic exercise of various durations. J Sports Sci. 1992;10(5):425–435. doi: 10.1080/02640419208729941 1331494

[65] Siegler JC , Hirscher K . Sodium bicarbonate ingestion and boxing performance. J Strength Cond Res. 2010;24(1):103–108. doi: 10.1519/JSC.0b013e3181a392b2 19625976

[66] Van Montfoort MCE , Van Dieren L , Hopkins WG , et al. Effects of ingestion of bicarbonate, citrate, lactate, and chloride on sprint running. Med Sci Sports Exerc. 2004;36(7):1239–1243. doi: 10.1249/01.MSS.0000132378.73975.25 15235332

[67] Artioli GG , Gualano B , Coelho DF , et al. Does sodium-bicarbonate ingestion improve simulated judo performance?. Int J Sport Nutr Exerc Metab. 2007;17(2):206–217. doi: 10.1123/ijsnem.17.2.206 17507744

[68] Bishop D , Claudius B . Effects of induced metabolic alkalosis on prolonged intermittent-sprint performance: Medicine & science in sports & exercise. Eur J Appl Physiol Occup. 2005;37(5):759–767. 10.1249/01.mss.0000161803.44656.3c 15870629

[69] Hobson RM , Harris RC , Martin D , et al. Effect of sodium bicarbonate supplementation on 2000-m rowing performance. Int J Sports Physiol Perform. 2014;9(1):139–144. doi: 10.1123/ijspp.2013-0086 23579002

[70] Leach NK , Hilton NP , Tinnion D , et al. Sodium bicarbonate ingestion in a fasted state improves 16.1-km cycling time-trial performance. Med Sci Sports Exerc. 2023;55(12):2299–2307. doi: 10.1249/MSS.0000000000003263 37535313

[71] Gurton WH , Matta GG , Gough LA , et al. Efficacy of sodium bicarbonate ingestion strategies for protecting blinding. Eur J Appl Physiol. 2022;122(12):2555–2563. doi: 10.1007/s00421-022-05031-0 36053364 PMC9613539

[72] Marticorena FM , Carvalho A , Oliveira LFD , et al. Nonplacebo controls to determine the magnitude of ergogenic interventions: a systematic review and meta-analysis. Med Sci Sports Exerc. 2021;53(8):1766–1777. doi: 10.1249/MSS.0000000000002635 33587551

[73] Cameron SL , McLay-Cooke RT , Brown RC , et al. Increased blood pH but not performance with sodium bicarbonate supplementation in elite rugby union players. Int J Sport Nutr Exerc Metab. 2010;20(4):307–321. doi: 10.1123/ijsnem.20.4.307 20739719

[74] Martin RAXJ , Hilton NP , Sparks SA , et al. The effects of enteric-coated sodium bicarbonate supplementation on 2 km rowing performance in female CrossFit® athletes. Eur J Appl Physiol. 2023;123(6):1191–1198. doi: 10.1007/s00421-023-05140-4 36705750 PMC10191925

[75] Goldfinch J , Mc Naughton L , Davies P . Induced metabolic alkalosis and its effects on 400-m racing time. Eur J Appl Physiol Occup Physiol. 1988;57(1):45–48. doi: 10.1007/BF00691236 2830108

[76] Christensen PM , Petersen MH , Friis SN , et al. Caffeine, but not bicarbonate, improves 6 min maximal performance in elite rowers. Appl Physiol Nutr Metab. 2014;39(9):1058–1063. doi: 10.1139/apnm-2013-0577 24999004

[77] Boegman S , Stellingwerff T , Shaw G , et al. The impact of individualizing sodium bicarbonate supplementation strategies on world-class rowing performance. Front Nutr [Internet]. 2020;7:138. doi: 10.3389/fnut.2020.00138 [cited 2025 Dec 10]. 33015117 PMC7509055

[78] Grgic J . Effects of sodium bicarbonate ingestion on measures of wingate test performance: a meta-analysis. J Am Nutr Assoc. 2022;41(1):1–10. doi: 10.1080/07315724.2020.1850370 33314967

[79] Douroudos II , Fatouros IG , Gourgoulis V , et al. Dose-related effects of prolonged NaHCO_3_ ingestion during high-intensity exercise. Med Sci Sports Exerc. 2006;38(10):1746–1753. doi: 10.1249/01.mss.0000230210.60957.67 17019296

[80] Mündel T . Sodium bicarbonate ingestion improves repeated high-intensity cycling performance in the heat. Temperature (Austin). 2018;5(4):343–347. doi: 10.1080/23328940.2018.1436393 30574526 PMC6298489

[81] Mero AA , Hirvonen P , Saarela J , et al. Effect of sodium bicarbonate and beta-alanine supplementation on maximal sprint swimming. J Int Soc Sports Nutr. 2013;10(1):52. doi: 10.1186/1550-2783-10-52 24215679 PMC4176133

[82] Zajac A , Cholewa J , Poprzecki S , et al. Effects of sodium bicarbonate ingestion on swim performance in youth athletes. J Sports Sci Med. 2009;8(1):45–50. 24150555 PMC3737792

[83] Zabala M , Requena B , Sánchez-Muñoz C , et al. Effects of sodium bicarbonate ingestion on performance and perceptual responses in a laboratory-simulated BMX cycling qualification series. J Strength Cond Res. 2008;22(5):1645–1653. doi: 10.1519/JSC.0b013e318181febe 18714219

[84] Zabala M , Peinado AB , Calderón FJ , et al. Bicarbonate ingestion has no ergogenic effect on consecutive all out sprint tests in BMX elite cyclists. Eur J Appl Physiol. 2011;111(12):3127–3134. doi: 10.1007/s00421-011-1938-8 21465247

[85] Peinado AB , Holgado D , Luque-Casado A , et al. Effect of induced alkalosis on performance during a field-simulated BMX cycling competition. J Sci Med Sport. 2019;22(3):335–341. doi: 10.1016/j.jsams.2018.08.010 30170952

[86] Álvarez-Herms J , Jornet K . Physiological data of kilian jornet during the victory of UTMB 2022: an exceptional report of maximal metabolical limits. Sports Med. 2024;54(12):3211–3214. doi: 10.1007/s40279-024-02091-4 39289331

[87] Burnley M , Jones AM . Oxygen uptake kinetics as a determinant of sports performance. Eur J Sport Sci. 2007;7(2):63–79. doi: 10.1080/17461390701456148

[88] Jones AM , Vanhatalo A . The ‘Critical power’ concept: applications to sports performance with a focus on intermittent high-intensity exercise. Sports Med. 2017;47(Suppl 1):65–78. doi: 10.1007/s40279-017-0688-0 28332113 PMC5371646

[89] George K , Maclaren D . The effect of induced alkalosis and acidosis on endurance running at an intensity corresponding to 4 mM blood lactate. Ergonomics. 1988;31(11):1639–1645. doi: 10.1080/00140138808966813 3229410

[90] Egger F , Meyer T , Such U , et al. Effects of sodium bicarbonate on high-intensity endurance performance in cyclists: a double-blind, randomized cross-over trial. PLoS One. 2014;9(12):e114729. doi: 10.1371/journal.pone.0114729 25494054 PMC4262454

[91] McNaughton L , Dalton B , Palmer G . Sodium bicarbonate can be used as an ergogenic aid in high-intensity, competitive cycle ergometry of 1 h duration. Eur J Appl Physiol Occup Physiol. 1999;80(1):64–69. doi: 10.1007/s004210050559 10367725

[92] Northgraves MJ , Peart DJ , Jordan CA , et al. Effect of lactate supplementation and sodium bicarbonate on 40-km cycling time trial performance. J Strength Cond Res. 2014;28(1):273–280. doi: 10.1519/JSC.0b013e3182986a4c 23660571

[93] Freis T , Hecksteden A , Such U , et al. Effect of sodium bicarbonate on prolonged running performance: a randomized, double-blind, cross-over study. PLoS One. 2017;12(8):e0182158. doi: 10.1371/journal.pone.0182158 28797049 PMC5552294

[94] Stephens TJ , McKenna MJ , Canny BJ , et al. Effect of sodium bicarbonate on muscle metabolism during intense endurance cycling. Med Sci Sports Exerc. 2002;34(4):614–621. 11932569 10.1097/00005768-200204000-00009

[95] Lassen TAH , Lindstrøm L , Lønbro S , et al. Increased performance in elite runners following individualized timing of sodium bicarbonate supplementation. Int J Sport Nutr Exerc Metab. 2021;31(6):453–459. doi: 10.1123/ijsnem.2020-0352 34470913

[96] Laursen PB , Francis GT , Abbiss CR , et al. Reliability of time-to-exhaustion versus time-trial running tests in runners. Med Sci Sports Exerc. 2007;39(8):1374–1379. doi: 10.1249/mss.0b013e31806010f5 17762371

[97] Falk Neto JH , Faulhaber M , Kennedy MD . The characteristics of endurance events with a variable pacing Profile—Time to embrace the concept of “Intermittent endurance Events”? Sports (Basel). 2024;12(6):164. doi: 10.3390/sports12060164 38921858 PMC11207974

[98] Theurel J , Lepers R . Neuromuscular fatigue is greater following highly variable versus constant intensity endurance cycling. Eur J Appl Physiol. 2008;103(4):461–468. doi: 10.1007/s00421-008-0738-2 18415118

[99] Potteiger JA , Webster MJ , Nickel GL , et al. The effects of buffer ingestion on metabolic factors related to distance running performance. Eur J Appl Physiol Occup Physiol. 1996;72(4):365–371. doi: 10.1007/BF00599698 8851907

[100] Carr AJ , McKay AKA , Burke LM , et al. Use of buffers in specific contexts: highly trained female athletes, extreme environments and combined buffering Agents-A narrative review. Sports Med. 2023;53(Suppl 1):25–48. doi: 10.1007/s40279-023-01872-7 37878211 PMC10721675

[101] Deb SK , Gough LA , Sparks SA , et al. Sodium bicarbonate supplementation improves severe-intensity intermittent exercise under moderate acute hypoxic conditions. Eur J Appl Physiol. 2018;118(3):607–615. doi: 10.1007/s00421-018-3801-7 29344729 PMC5805802

[102] Deb SK , Gough LA , Sparks SA , et al. Determinants of curvature constant (W') of the power duration relationship under normoxia and hypoxia: the effect of pre-exercise alkalosis. Eur J Appl Physiol. 2017;117(5):901–912. doi: 10.1007/s00421-017-3574-4 28280973 PMC5388723

[103] Shannon ES , Sparks SA , McNaughton LR , et al. The effect of sodium bicarbonate mini-tablets in a carbohydrate hydrogel on prolonged high-intensity cycling performance and metabolism in acute normobaric hypoxia. Eur J Appl Physiol [Internet]. 2026;126(5):2911–2925. doi: 10.1007/s00421-025-06069-6. cited 2026 Jan. 41483411 PMC13236792

[104] Gough LA , Brown D , Deb SK , et al. The influence of alkalosis on repeated high-intensity exercise performance and acid–base balance recovery in acute moderate hypoxic conditions. Eur J Appl Physiol. 2018;118(12):2489–2498. doi: 10.1007/s00421-018-3975-z 30196448 PMC6244684

[105] Saunders B , Sale C , Harris RC , et al. Effect of sodium bicarbonate and beta-alanine on repeated sprints during intermittent exercise performed in hypoxia. Int J Sport Nutr Exerc Metab. 2014;24(2):196–205. doi: 10.1123/ijsnem.2013-0102 24225816

[106] Flinn S , Herbert K , Graham K , et al. Differential effect of metabolic alkalosis and hypoxia on high-intensity cycling performance. J Strength Condit Res. 2014;28(1):2852–2858. doi: 10.1519/JSC.0000000000000489 24983849

[107] Adams RP , Welch HG . Oxygen uptake, acid-base status, and performance with varied inspired oxygen fractions. J Appl Physiol. 1980;49(5):863–868. doi: 10.1152/jappl.1980.49.5.863 6776081

[108] Gore CJ , Hahn AG , Aughey RJ , et al. Live high: train low increases muscle buffer capacity and submaximal cycling efficiency. Acta Physiol Scand. 2001;173(3):275–286. doi: 10.1046/j.1365-201X.2001.00906.x 11736690

[109] Kayser B , Ferretti G , Grassi B , et al. Maximal lactic capacity at altitude: effect of bicarbonate loading. J Appl Physiol. 1993;75(3):1070–1074. doi: 10.1152/jappl.1993.75.3.1070 8226513

[110] Kozak-Collins K , Burke EF , Schoene RB . Sodium bicarbonate ingestion does not improve performance in women cyclists. Med Sci Sports Exercise. 1994;26:1510–1515. 7869886

[111] Hausswirth C , Bigard A , Lepers R , et al. Sodium citrate ingestion and muscle performance in acute hypobaric hypoxia. Eur J Appl Occup Physiol. 1995;71:362–368. doi: 10.1007/BF00240418 8549581

[112] Savoie FA , Asselin A , Goulet ED . Comparison of sodium chloride tablets-induced, sodium chloride solution-induced, and glycerol-induced hyperhydration on fluid balance responses in healthy men. The Journal of Strength & Conditioning Research. 2016;30(10):2880–2891. doi: 10.1519/JSC.0000000000001371 26849790

[113] Sims ST , Rehrer NJ , Bell ML , et al. Preexercise sodium loading aids fluid balance and endurance for women exercising in the heat. J Appl Physiol. 2007;103:534–541. doi: 10.1152/japplphysiol.01203.2006 17463297

[114] Gough LA , Williams JJ , Newbury JW , et al. The effects of sodium bicarbonate supplementation at individual time-to-peak blood bicarbonate on 4-km cycling time trial performance in the heat. Eur J Sport Sci. 2022;22(12):1856–1864. doi: 10.1080/17461391.2021.1998644 34704539

[115] Katagiri A , Kitadai Y , Miura A , et al. Sodium bicarbonate ingestion mitigates the heat-induced hyperventilation and reduction in cerebral blood velocity during exercise in the heat. J Appl Physiol (1985). 2021;131(5):1617–1628. doi: 10.1152/japplphysiol.00261.2021 34590911

[116] Kupcis PD , Slater GJ , Pruscino CL , et al. Influence of sodium bicarbonate on performance and hydration in lightweight rowing. Int J Sports Physiol Perform. 2012;7:11–18. doi: 10.1123/ijspp.7.1.11 21941012

[117] Suvi S , Mooses M , Timpmann S , et al. Impact of sodium citrate ingestion during recovery after dehydrating exercise on rehydration and subsequent 40-km cycling time-trial performance in the heat. Appl Physiol Nutr Metab. 2018;43(6):571–579. doi: 10.1139/apnm-2017-0584 29324186

[118] Timpmann S , Burk A , Medijainen L , et al. Dietary sodium citrate supplementation enhances rehydration and recovery from rapid body mass loss in trained wrestlers. Appl Physiol Nutr Metab. 2013;36(6):1028–1037. 10.1139/h2012-089 22871128

[119] Masoud AA , Li Z , Deyhle MR , et al. Acute sodium bicarbonate supplementation reduces the increase in markers of acute kidney injury during physical work in the heat. Eur J Appl Physiol. 2025;125:3619–3630. doi: 10.1007/s00421-025-05850-x 40542865

[120] Aktitiz S , Koşar ŞN , Turnagöl HH . Effects of acute and multi-day low-dose sodium bicarbonate intake on high-intensity endurance exercise performance in Male recreational cyclists. Eur J Appl Physiol. 2024;124(7):2111–2122. doi: 10.1007/s00421-024-05434-1 38421429 PMC11199215

[121] Durkalec-Michalski K , Nowaczyk PM , Kamińska J , et al. The interplay between bicarbonate kinetics and gastrointestinal upset on ergogenic potential after sodium bicarbonate intake: a randomized double-blind placebo-controlled trial. Sci Rep. 2023;13(1):7081. doi: 10.1038/s41598-023-34343-0 37127791 PMC10151363

[122] McNaughton LR , Gough L , Deb S , et al. Recent developments in the use of sodium bicarbonate as an ergogenic aid. Curr Sports Med Rep. 2016;15(4):233–244. doi: 10.1249/JSR.0000000000000283 27399820

[123] Sparks A , Williams E , Robinson A , et al. Sodium bicarbonate ingestion and individual variability in time-to-peak pH. Res Sports Med. 2017;25(1):58–66. doi: 10.1080/15438627.2016.1258645 27934546

[124] Correia-Oliveira CR , Lopes-Silva JP , Bertuzzi R , et al. Acidosis, but not alkalosis, affects anaerobic metabolism and performance in a 4-km time trial. Med Sci Sports Exerc. 2017;49(9):1899–1910. doi: 10.1249/MSS.0000000000001295 28398947

[125] Callahan MJ , Parr EB , Hawley JA , et al. Single and combined effects of beetroot crystals and sodium bicarbonate on 4-km cycling time trial performance. Int J Sport Nutr Exerc Metab. 2017;27(3):271–278. doi: 10.1123/ijsnem.2016-0228 27834492

[126] Gurton WH , King DG , Ranchordas MK , et al. Enhancing exercise performance and recovery through sodium bicarbonate supplementation: introducing the ingestion recovery framework. Eur J Appl Physiol. 2024;124(11):3175–3190. doi: 10.1007/s00421-024-05578-0 39177769 PMC11519211

[127] Gough L , Rimmer S , Osler C , et al. Ingestion of sodium bicarbonate (NaHCO_3_) following a fatiguing bout of exercise accelerates post-exercise acid-base balance recovery and improves subsequent high-intensity cycling time to exhaustion. Int J Sport Nutr Exercise Metab. 2017;27:1–25. doi: 10.1123/ijsnem.2017-0065 28530505

[128] Gurton W , Macrae H , Gough L , et al. Effects of post-exercise sodium bicarbonate ingestion on acid-base balance recovery and time-to-exhaustion running performance: a randomised crossover trial in recreational athletes. Appl Physiol Nutr Metab. 2021;46(9):1111–1118. doi: 10.1139/apnm-2020-1120 33730517

[129] Peart DJ , McNaughton LR , Midgley AW , et al. Pre-exercise alkalosis attenuates the heat shock protein 72 response to a single-bout of anaerobic exercise. J Sci Med Sport. 2011;14(5):435–440. doi: 10.1016/j.jsams.2011.03.006 21498114

[130] Thomas C , Delfour‐Peyrethon R , Lambert K , et al. The effect of pre-exercise alkalosis on lactate/pH regulation and mitochondrial respiration following sprint-interval exercise in humans. Front Physiol [Internet]. 2023;14: doi: 10.3389/fphys.2023.1073407 [cited 2023 Mar 8]. PMC9911540 36776968

[131] Peart DJ , Kirk RJ , Madden LA , et al. Implications of a pre-exercise alkalosis-mediated attenuation of HSP72 on its response to a subsequent bout of exercise. Amino Acids. 2016;48(2):499–504. doi: 10.1007/s00726-015-2103-1 26433893

[132] Percival ME , Martin BJ , Gillen JB , et al. Sodium bicarbonate ingestion augments the increase in PGC-1α mRNA expression during recovery from intense interval exercise in human skeletal muscle. J Appl Physiol. 2015;119(11):1303–1312. doi: 10.1152/japplphysiol.00048.2015 26384407 PMC4669344

[133] Dalle S , Smet SD , Geuns W , et al. Effect of stacked sodium bicarbonate loading on repeated all-out exercise. Int J Sports Med. 2019;40(11):711–716. doi: 10.1055/a-0978-5139 31434137

[134] Pruscino CL , Ross MLR , Gregory JR , et al. Effects of sodium bicarbonate, caffeine, and their combination on repeated 200-m freestyle performance. Int J Sport Nutr Exerc Metab. 2008;18(2):116–130. doi: 10.1123/ijsnem.18.2.116 18458356

[135] Gurton WH , Gough LA , Lynn A , et al. Effect of oral and topical sodium bicarbonate on functional recovery and soccer-specific performance after exercise-induced muscle damage: a randomized, double-blind, placebo-controlled study. Nutrients. 2025;17(21):3383. doi: 10.3390/nu17213383 41228456 PMC12610355

[136] Battazza RA , Kalytczak MM , Leite CDFC , et al. Effect of sodium bicarbonate supplementation on muscle performance and muscle damage: a double blind, randomized crossover study. J Diet Suppl. 2023;20(5):689–705. doi: 10.1080/19390211.2022.2090478 35758017

[137] Venier S , Grgic J , Mikulic P . Acute enhancement of jump performance, muscle strength, and power in resistance-trained men after consumption of caffeinated chewing gum. Int J Sports Physiol Perform. 2019;14(10):1415–1421. doi: 10.1123/ijspp.2019-0098 30958062

[138] Saunders B , Elliott-Sale K , Artioli GG , et al. β-alanine supplementation to improve exercise capacity and performance: a systematic review and meta-analysis. Br J Sports Med. 2017;51(8):658–669. doi: 10.1136/bjsports-2016-096396 27797728

[139] Edge J , Bishop D , Goodman C . Effects of chronic NaHCO_3_ ingestion during interval training on changes to muscle buffer capacity, metabolism, and short-term endurance performance. J Appl Physiol (1985). 2006;101(3):918–925. doi: 10.1152/japplphysiol.01534.2005 16627675

[140] Bishop DJ , Thomas C , Moore-Morris T , et al. Sodium bicarbonate ingestion prior to training improves mitochondrial adaptations in rats. Am J Physiol Endocrinol Metab. 2010;299(2):E225–233. doi: 10.1152/ajpendo.00738.2009 20484007

[141] Wang J , Qiu J , Yi L , et al. Effect of sodium bicarbonate ingestion during 6 weeks of HIIT on anaerobic performance of college students. J Int Soc Sports Nutr. 2019;16(1):18. doi: 10.1186/s12970-019-0285-8 30987663 PMC6466775

[142] Siegler JC , Marshall PWM , Finn H , et al. Acute attenuation of fatigue after sodium bicarbonate supplementation does not manifest into greater training adaptations after 10-weeks of resistance training exercise. PLoS One. 2018;13(5):e0196677. doi: 10.1371/journal.pone.0196677 29718968 PMC5931633

[143] Driller MW , Gregory JR , Williams AD , et al. The effects of chronic sodium bicarbonate ingestion and interval training in highly trained rowers. Int J Sport Nutr Exerc Metab. 2013;23(1):40–47. doi: 10.1123/ijsnem.23.1.40 22899814

[144] Ament W , Verkerke GJ . Exercise and fatigue. Sports Med. 2009;39(5):389–422. doi: 10.2165/00007256-200939050-00005 19402743

[145] Maughan RJ , Burke LM , Dvorak J , et al. IOC consensus statement: dietary supplements and the high-performance athlete. Br J Sports Med. 2018;52(7):439–455. doi: 10.1136/bjsports-2018-099027 29540367 PMC5867441

[146] Ducker KJ , Dawson B , Wallman KE . Effect of beta alanine and sodium bicarbonate supplementation on repeated-sprint performance. J Strength Cond Res. 2013;27(12):3450–3460. doi: 10.1519/JSC.0b013e31828fd310 23524361

[147] Siegler JC , Gleadall-Siddall DO . Sodium bicarbonate ingestion and repeated swim sprint performance. J Strength Cond Res. 2010;24(11):3105–3111. doi: 10.1519/JSC.0b013e3181f55eb1 20881504

[148] Wilkes D , Gledhill N , Smyth R . Effect of acute induced metabolic alkalosis on 800-m racing time. Med Sci Sports Exerc. 1983;15(4):277–280. doi: 10.1249/00005768-198315040-00004 6312244

[149] Carr AJ , Slater GJ , Gore CJ , et al. Reliability and effect of sodium bicarbonate: buffering and 2000-m rowing performance. Int J Sports Physiol Perform. 2012;7(2):152–160. doi: 10.1123/ijspp.7.2.152 22634964

[150] Gurton WH , Dabin L , Marshall S . No effect of individualized sodium bicarbonate supplementation on 200-m or 400-m freestyle-swimming time-trial performance in well-trained athletes. 2024; [cited 2025 Sept 1]. doi: 10.1123/ijspp.2023-0535 39708790

[151] Tan F , Polglaze T , Cox G , et al. Effects of induced alkalosis on simulated match performance in elite female water polo players. Int J Sport Nutr Exerc Metab. 2010;20(3):198–205. doi: 10.1123/ijsnem.20.3.198 20601737

[152] Felippe LC , Lopes-Silva JP , Bertuzzi R , et al. Separate and combined effects of caffeine and sodium-bicarbonate intake on judo performance. Int J Sports Physiol Perform. 2016;11(2):221–226. doi: 10.1123/ijspp.2015-0020 26182440

[153] Wu C-L , Shih M-C , Yang C-C , et al. Sodium bicarbonate supplementation prevents skilled tennis performance decline after a simulated match. J Int Soc Sports Nutr. 2010;7:33. doi: 10.1186/1550-2783-7-33 20977701 PMC2978121

[154] Salt in your diet [Internet] nhs.uk. 2022. [cited 2026 Jan 16]. Available from: 2022. https://www.nhs.uk/live-well/eat-well/food-types/salt-in-your-diet/

[155] Hayakawa Y , Komaki H , Minatoguchi S , et al. High-salt intake accelerates functional and histological renal damage associated with renal tissue overexpression of (pro)renin receptors and AT1 receptors in spontaneously hypertensive rats. Clin Exp Nephrol. 2020;24(7):582–589. doi: 10.1007/s10157-020-01888-7 32246289

[156] Ohta Y , Tsuchihashi T , Kiyohara K , et al. High salt intake promotes a decline in renal function in hypertensive patients: a 10-year observational study. Hypertens Res. 2013;36(2):172–176. doi: 10.1038/hr.2012.155 23051657

[157] Sostaric SM , Skinner SL , Brown MJ , et al. Alkalosis increases muscle K^+^ release, but lowers plasma [K^+^] and delays fatigue during dynamic forearm exercise. J. Physiol. 2006;570(Pt 1):185–205. doi: 10.1113/jphysiol.2005.094615 PMC1464289 16239279

[158] Gurton WH , Greally J , Chudzikiewicz K , et al. Beneficial effects of oral and topical sodium bicarbonate during a battery of team sport-specific exercise tests in recreationally trained Male athletes. J Int Soc Sports Nutr. 2023;20(1):2216678. doi: 10.1080/15502783.2023.2216678 37227399 PMC10215010

[159] Lancha Junior AH , Painelli V , de S , et al. Nutritional strategies to modulate intracellular and extracellular buffering capacity during high-intensity exercise. Sports Med. 2015;45(Suppl 1):S71–81. doi: 10.1007/s40279-015-0397-5 26553493 PMC4672007

[160] Harris RC , Tallon MJ , Dunnett M , et al. The absorption of orally supplied β-alanine and its effect on muscle carnosine synthesis in human vastus lateralis. Amino Acids. 2006;30(3):279–289. doi: 10.1007/s00726-006-0299-9 16554972

[161] Dunnett M , Harris RC . Influence of oral beta-alanine and L-histidine supplementation on the carnosine content of the gluteus medius. Equine Vet J. 1999;Suppl(30):499–504. doi: 10.1111/j.2042-3306.1999.tb05273.x 10659307

[162] Hultman E , Sahlin K . Acid-base balance during exercise. Exerc Sport Sci Rev. 1980;8:41–128. 7016549

[163] Saunders B , DE Salles Painelli V , DE Oliveira LF , et al. Twenty-four weeks of β-Alanine supplementation on carnosine content, related genes, and exercise. Med Sci Sports Exerc. 2017;49(5):896–906. doi: 10.1249/MSS.0000000000001173 28157726

[164] Baguet A , Reyngoudt H , Pottier A , et al. Carnosine loading and washout in human skeletal muscles. J Appl Physiol (1985). 2009;106(3):837–842. doi: 10.1152/japplphysiol.91357.2008 19131472

[165] Trexler ET , Smith-Ryan AE , Stout JR , et al. International society of sports nutrition position stand: beta-alanine. J Int Soc Sports Nutr. 2015;12:30. doi: 10.1186/s12970-015-0090-y 26175657 PMC4501114

[166] Perim P , Marticorena FM , Ribeiro F , et al. Can the skeletal muscle carnosine response to beta-alanine supplementation be optimized? Front Nutr. 2019;6:135. doi: 10.3389/fnut.2019.00135 31508423 PMC6718727

[167] Gough LA , Rimmer S , Osler CJ , et al. Ingestion of sodium bicarbonate (NaHCO_3_) following a fatiguing bout of exercise accelerates postexercise acid-base balance recovery and improves subsequent high-intensity cycling time to exhaustion. Int J Sport Nutr Exerc Metab. 2017;27(5):429–438. doi: 10.1123/ijsnem.2017-0065 28530505

[168] Maciejewski H , Bourdin M , Féasson L , et al. Muscle MCT4 content is correlated with the lactate removal ability during recovery following all-out supramaximal exercise in highly-trained rowers. Front Physiol. 2016;7:223. doi: 10.3389/fphys.2016.00223 27375499 PMC4901069

[169] Stellingwerff T , Anwander H , Egger A , et al. Effect of two β-alanine dosing protocols on muscle carnosine synthesis and washout. Amino Acids. 2012;42(6):2461–2472. doi: 10.1007/s00726-011-1054-4 21847611

[170] Saunders B , Franchi M , de Oliveira LF , et al. 24-Week β-alanine ingestion does not affect muscle taurine or clinical blood parameters in healthy males. Eur J Nutr. 2020;59(1):57–65. doi: 10.1007/s00394-018-1881-0 30552505

[171] Dolan E , Swinton PA , Painelli V de S , et al. A systematic risk assessment and meta-analysis on the use of oral β-Alanine supplementation. Adv Nutr. 2019;10(3):452–463. doi: 10.1093/advances/nmy115 30980076 PMC6520041

[172] Newbury JW , Cole M , Kelly AL , et al. The time to peak blood bicarbonate (HCO_3–_), pH, and the strong ion difference (SID) following sodium bicarbonate (NaHCO_3_) ingestion in highly trained adolescent swimmers. PLoS One. 2021;16(7):e0248456. doi: 10.1371/journal.pone.0248456 34197456 PMC8248647

[173] Hobson RM , Saunders B , Ball G , et al. Effects of β-alanine supplementation on exercise performance: a meta-analysis. Amino Acids. 2012;43(1):25–37. doi: 10.1007/s00726-011-1200-z 22270875 PMC3374095

[174] Hadzic M , Eckstein ML , Schugardt M . The impact of sodium bicarbonate on performance in response to exercise duration in athletes: a systematic review. J Sports Sci Med. 2019;18(2):271–281. 31191097 PMC6544001

[175] Bellinger PM , Howe ST , Shing CM , et al. Effect of combined β-Alanine and SodiumBicarbonate supplementation on cycling performance. Med Sci Sports Exercise. 2012;44(8):1545–1551. doi: 10.1249/MSS.0b013e31824cc08d 22330016

[176] Sale C , Saunders B , Hudson S , et al. Effect of β-Alanine plus sodium bicarbonate on high-intensity cycling capacity. Med Sci Sports Exercise. 2011;43(10):1972–1978. doi: 10.1249/MSS.0b013e3182188501 21407127

[177] Tobias G , Benatti FB , de Salles Painelli V , et al. Additive effects of beta-alanine and sodium bicarbonate on upper-body intermittent performance. Amino Acids. 2013;45(2):309–317. doi: 10.1007/s00726-013-1495-z 23595205 PMC3714561

[178] Curran-Bowen T , Guedes da Silva A , Barreto G , et al. Sodium bicarbonate and beta-alanine supplementation: is combining both better than either alone? A systematic review and meta-analysis. Biol Sport. 2024;41(3):79–87. doi: 10.5114/biolsport.2024.132997 38952910 PMC11167468

[179] Gurton WH , Matta GG , Gough LA , et al. Sodium bicarbonate and time-to-exhaustion cycling performance: a retrospective analysis exploring the mediating role of expectation. Sports Med Open. 2023;9(1):65. doi: 10.1186/s40798-023-00612-5 37523028 PMC10390418

[180] McClung M , Collins D . Because I know it will!”: placebo effects of an ergogenic aid on athletic performance. J Sport Exerc Psychol. 2007;29(3):382–394. doi: 10.1123/jsep.29.3.382 17876973

[181] Zagatto AM , Lopes VHF , Dutra YM , et al. Sodium bicarbonate induces alkalosis, but improves high-intensity cycling performance only when participants expect a beneficial effect: a placebo and nocebo study. Eur J Appl Physiol. 2024;124(5):1367–1380. doi: 10.1007/s00421-023-05368-0 38032386

[182] Durkalec-Michalski K , Zawieja EE , Zawieja BE , et al. The gender dependent influence of sodium bicarbonate supplementation on anaerobic power and specific performance in female and Male wrestlers. Sci Rep. 2020;10(1):1878. 32024852 10.1038/s41598-020-57590-x PMC7002590

[183] Higgins MF , Shabir A . Expectancy of ergogenicity from sodium bicarbonate ingestion increases high-intensity cycling capacity. Appl Physiol Nutr Metab. 2016;41(4):405–410. doi: 10.1139/apnm-2015-0523 26863442

[184] Grgic J , Grgic I , Del Coso J , et al. Effects of sodium bicarbonate supplementation on exercise performance: an umbrella review. J Int Soc Sports Nutr. 2021;18(1):71. doi: 10.1186/s12970-021-00469-7 34794476 PMC8600864

[185] Hegge AM , Bucher E , Ettema G , et al. Gender differences in power production, energetic capacity and efficiency of elite cross-country skiers during whole-body, upper-body, and arm poling. Eur J Appl Physiol. 2016;116(2):291–300. doi: 10.1007/s00421-015-3281-y 26476546

[186] Porter MM , Stuart S , Boij M , et al. Capillary supply of the tibialis anterior muscle in young, healthy, and moderately active men and women. J Appl Physiol (1985). 2002;92(4):1451–1457. doi: 10.1152/japplphysiol.00744.2001 11896009

[187] Simoneau JA , Bouchard C . Human variation in skeletal muscle fiber-type proportion and enzyme activities. Am J Physiol. 1989;257(4 Pt 1):E567–572. doi: 10.1152/ajpendo.1989.257.4.E567 2529775

[188] Green HJ , Fraser IG , Ranney DA . Male and female differences in enzyme activities of energy metabolism in vastus lateralis muscle. J Neurol Sci. 1984;65(3):323–331. doi: 10.1016/0022-510X(84)90095-9 6238135

[189] Russ DW , Lanza IR , Rothman D , et al. Sex differences in glycolysis during brief, intense isometric contractions. Muscle Nerve. 2005;32(5):647–655. doi: 10.1002/mus.20396 16025523

[190] Durkalec-Michalski K , Nowaczyk PM , Saunders B , et al. Sex-dependent responses to acute sodium bicarbonate different dose treatment: a randomized double-blind crossover study. J Sci Med Sport. 2025;28(2):154–165. doi: 10.1016/j.jsams.2024.08.209 39306577

[191] McNaughton LR , Ford S , Newbold C . Effect of sodium bicarbonate ingestion on high intensity exercise in moderately trained women. J Strength Condit Res. 1997;11(2):98.

[192] Delextrat A , Mackessy S , Arceo-Rendon L , et al. Effects of three-day serial sodium bicarbonate loading on performance and physiological parameters during a simulated basketball test in female university players. Int J Sport Nutr Exerc Metab. 2018;28(5):547–552. doi: 10.1123/ijsnem.2017-0353 29345173

